# Wavelet brain angiography suggests arteriovenous pulse wave phase locking

**DOI:** 10.1371/journal.pone.0187014

**Published:** 2017-11-15

**Authors:** William E. Butler

**Affiliations:** Massachusetts General Hospital, Neurosurgical Service, Boston, Massachusetts 02114, United States of America; Ehime University Graduate School of Medicine, JAPAN

## Abstract

When a stroke volume of arterial blood arrives to the brain, the total blood volume in the bony cranium must remain constant as the proportions of arterial and venous blood vary, and by the end of the cardiac cycle an equivalent volume of venous blood must have been ejected. I hypothesize the brain to support this process by an extraluminally mediated exchange of information between its arterial and venous circulations. To test this I introduce wavelet angiography methods to resolve single moving vascular pulse waves (PWs) in the brain while simultaneously measuring brain pulse motion. The wavelet methods require angiographic data acquired at significantly faster rate than cardiac frequency. I obtained these data in humans from brain surface optical angiograms at craniotomy and in piglets from ultrasound angiograms via cranial window. I exploit angiographic time of flight to resolve arterial from venous circulation. Initial wavelet reconstruction proved unsatisfactory because of angiographic motion alias from brain pulse motion. Testing with numerically simulated cerebral angiograms enabled the development of a vascular PW cine imaging method based on cross-correlated wavelets of mixed high frequency and high temporal resolution respectively to attenuate frequency and motion alias. Applied to the human and piglet data, the method resolves individual arterial and venous PWs and finds them to be phase locked each with separate phase relations to brain pulse motion. This is consistent with arterial and venous PW coordination mediated by pulse motion and points to a testable hypothesis of a function of cerebrospinal fluid in the ventricles of the brain.

## Introduction

The forces behind brain pulsations arise in the heart, which pumps blood to the brain in a sequence of stroke volumes. When an arterial stroke volume arrives to the intracranial compartment, a hemodynamic variation spreads through the cerebrovascular tree and induces brain motion. There are constraints to this process, and I hypothesize that these constraints oblige the brain to handle an arriving stroke volume with an orderly pattern of brain pulse motion, arterial pulse waves (PWs), and venous PWs. To test this hypothesis, I develop wavelet imaging methods to time resolve the joint cardiac frequency (CF) processes of brain motion and hemodynamic variation.

An example constraint is that an arriving arterial stroke volume ought to displace from within the bony cranium an approximately equal volume of venous blood across the cardiac cycle [1]. I hypothesize that to coordinate this displacement, an arriving stroke volume of arterial blood somehow induces a transfer of information across extravascular spaces to the venous circulation. The transfer of information may be mediated by solid brain tissue, by cerebrospinal fluid (CSF) including in the ventricles of the brain, or cooperatively by both. I hypothesize that the net transfer of information from the arterial side to the venous side enables the measured ejection from the cranium of an appropriate volume of venous blood coupled with an appropriate quantity of kinetic vascular energy. This way in a given cardiac cycle there is a match of the volumes and kinetic energies after frictional losses of blood moving in and out of the cranium. It is unlikely that there is sufficient CF information exchanged through the capillaries to coordinate this balance because experimental cranial window microscopy studies have not found pulsatile velocity changes of red blood cells in brain capillaries [2].

This paper focuses then on the topic of tissue pulse motion and its interaction with arterial and venous PWs within that tissue. A related manuscript explores a potential contribution to the information transfer by CSF in the ventricles of the brain [3].

Those CF phenomena that show coherence, meaning relative spatial uniformity of CF phase, are defined in this paper to represent a PW. The modifier “pulse” signifies CF timing.

Whether an arriving arterial stroke volume ejects a matching volume of venous blood by simple conservation of mass, meaning for example that an arterial wall diameter expansion mechanically causes venous wall contraction, or whether active regulatory systems participate is beyond the scope of this paper. In either case, the hypothesis that there is coordination among tissue pulse motion and arterial and venous PWs experimentally predicts that

- Arterial blood in the brain forms a PW.
- Venous blood in the brain forms a PW.
- There is CF brain motion.
- These separately resolvable CF phenomena maintain coordinated phase relations.

Wavelets are mathematical objects that may enable the time-resolved extraction of CF phenomena. If the sampling rate is fast enough, wavelet methods should allow the deconstruction of events at a fine temporal resolution within each given of a sequence of cardiac cycles. Wavelet methods require that the underlying non-sparse angiographic data be acquired at a sampling rate significantly higher than CF, as governed by the sampling theorem of Nyqvist, Shannon, and Kotelnikov [4–7]. I report here data from human brain surface optical angiography and from piglet cranial window ultrasound angiography. Both of these angiographic methods allow at 30 Hz the acquisition of angiographic images of a passing bolus of contrast simultaneous with the observation of brain motion.

To classify a vascular PW as arterial or venous, I exploit the time of flight of a sharply delivered angiographic bolus, and assume vascular PWs that occur early to be arterial and those that occur late to be venous.

### Cardiac gated imaging methods

Under certain circumstances, CF phenomena in the brain may be imaged by those computed tomography (CT) and magnetic resonance imaging (MRI) methods that rely on cardiac gating [8–11]. In that approach, the timing of individual images is matched to an electrocardiogram (ECG) signal to enable the eventual interpolation of images within an averaged cardiac cycle [12]. Unlike the methods reported here, cardiac gated imaging methods do not image directly a chain of events internal to a given cardiac cycle. Instead, cardiac gating relies on the assumption that all heart beats are alike. Similarly, gated image sequences cannot capture variant phenomena that span from one heart beat to the next. Experimental methods that focus on physiological signals such as blood pressure and intracranial pressure (ICP) have the temporal resolution to register single CF events but since they do not offer images they are limited in their ability to portray the spatial basis of causal relations [13–15].

## Materials and methods

The human brain surface optical and piglet cranial window ultrasound angiography methods employed here share the properties of (1) capturing angiographic images at a rate significantly greater than CF, (2) permitting the concurrent measurement of brain motion, and (3) offering angiographic time of flight (ATOF) to separate arterial from venous circulation.

### Human brain surface optical angiography

#### Subjects

I inspected archived optical angiograms of 11 personally treated aneurysm craniotomy undergoing craniotomy for a cerebral aneurysm to reduce the risk of aneurysmal re-hemorrhage. These human patients were managed according to clinical conventions [16]. No aspect of their care was altered for the purpose of this study. There is no attempt in this study to comment on the clinical value of intraoperative human brain surface optical angiography, nor is there any intent to suggest whether or not the methods developed here have any potential for clinical utility. The human portion of this study is limited to the post-hoc analysis of archived data routinely collected in the course of clinical care. The data have no individually identifying features. Hence, Partners Human Research Committee in approving this research waived the requirement to approach the subjects for consent.

Some characteristics of the intraoperative optical angiograms as analyzed here that promote their clinical value do not promote their scientific value for wavelet analysis. For example, since optical angiography is a line of sight technique, to fully visualize all of the vasculature in the vicinity of an aneurysm, in most of my operated cases I move the microscope actively or manually manipulate tissues during the angiogram in order to see behind them. In addition, since the vasculature of interest is arterial, optical angiographic recording may be halted before entry into the venous phase. Of those, optical angiograms in four human subjects (designated h1-h4) met criteria for wavelet analysis. Their mean age at the time of surgery was 64.5 years (range 59 to 76 years). There was no optical angiogram where wavelet analysis was commenced but completed.

#### Procedure

At craniotomy under general anesthesia, the aneurysm and associated vessels are exposed with microsurgical technique with an operating microscope that is equipped to perform intraoperative indocyanine green angiography (Carl Zeiss Surgical Microscope OPMI Pentero INFRARED 800, Carl Zeiss Meditec AG, Jena, Germany). Generally these aneurysms lay in clefts between the lobes and fissures of the brain, and the surrounding brain surface is neocortex formatted as gyri and sulci (Fig A of S1 File). A temporary clip is applied to the aneurysm’s major feeding artery, the neck of the aneurysm undergoes final dissection, a clip is applied across the neck to exclude the aneurysm from blood flow while not including apart of the parent artery in the clip since that would impair blood flow through it. The temporary clip on the major feeding artery is removed.

**Fig 1:**
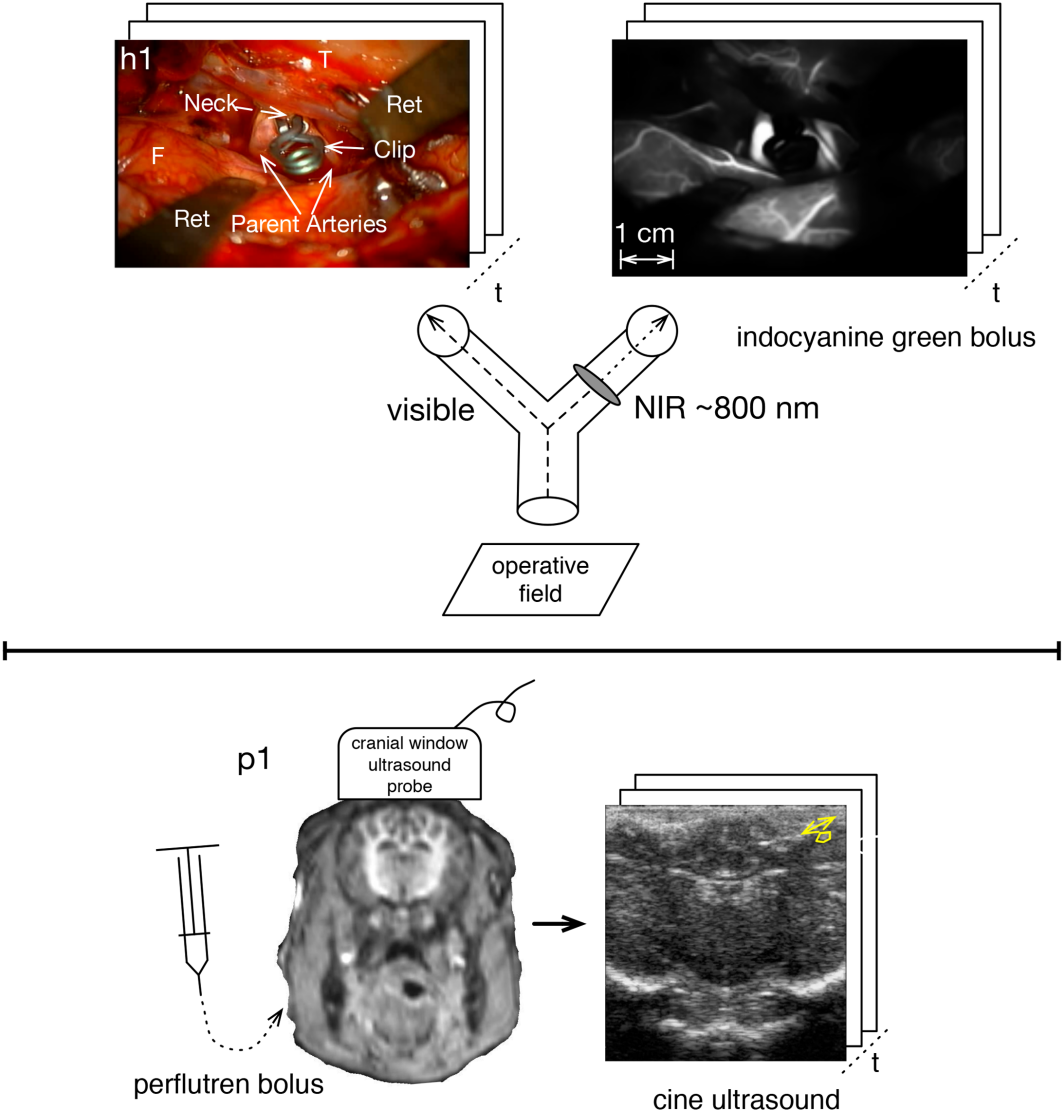
Angiography sampled faster than cardiac rate. Upper panel is human brain surface optical angiography with simultaneous visible recording via a beam splitter. The aneurysm is of the right middle cerebral artery, where F = frontal lobe, T = temporal lobe, and Ret = retractors. The lower panel is piglet ultrasound angiography via cranial window.

At this point the intraoperative optical angiogram is performed to confirm that the clipped aneurysm no longer fills with blood and that the associated vessels have not been inadvertently occluded by the clipping [17–21]. On signal, the anesthesiologist injects intravenously 25 mg of indocyanine green (ICG) and the surgeon presses a sterilely covered control button on the operating microscope to trigger the video recording through the microscope of two optical channels via a beam splitter. One channel records visible wavelength, and other contains a near infrared (NIR) pass filter that records the ICG fluorescence and thereby yields NIR ICG video-angiogram (Fig 1). The two video recordings, captured each at 30 Hz, are prepared for analysis as described in Section A-B of S1 File. The image spatial dimensions and sampling characteristics are presented in Table A of S1 File.

### Piglet cranial window ultrasound angiography

While ultrasound can image at a faster than cardiac rate, the blockage of sound by the skull bone impedes its sensitivity for this purpose [22]. In the piglet model reported here, the angiographic ultrasound imaging is through a cranial window of a passing bolus of perflutren, an ultrasound contrast agent [23]. The same ultrasound cine images allow the measurement of tissue motion by the tracking of translations of discrete brain structures (Fig 1).

#### Subjects

Ultrasound angiography though a cranial window was performed in three piglets weighing 10 kg (designated p1-p3) with approval by Partners Subcommittee on Research Animal Care. In one of them, the cranial window experiment was performed in an MRI suite, offering near simultaneous high anatomic resolution.

#### Procedure

Prior to the piglet experiments, the signal characteristics of the contrast agent, perflutren, was tested in dynamic phantoms and found to be suitable for these purposes. This is further described in Section B-A of S1 File.

Under the care of a veterinarian, the piglets were premedicated with intramuscular xylozine 2 mg/kg then general anesthesia was induced with an intramuscular tiletamine/zolazepam combination and maintained with inhalational isoflurane. ECG leads, a peripheral pulse oximetry monitor, and a femoral vein line were applied to each of the three anesthetized piglets with the animals positioned supine. Each animal was then turned prone, and with the head rigidly held above the level of the heart a craniectomy centered on the coronal suture was performed of the minimal size required to allow direct application of the ultrasound probe to the dura. The ultrasound probe was held firmly in place during imaging by a rigid mechanical arm (lower panel of Fig 1).

After acquiring baseline cine images, an activated vial containing 1.2 million microspheres was manually injected sharply into the femoral vein catheter and cine images of the intracranial cavity were recorded until the bolus of contrast had passed and steady state achieved (approximately 20 seconds). In one experiment, the animal was transported immediately after ultrasound imaging to a 3-Tesla MRI unit. The magnetic resonance images serve to confirm anatomic orientation of the ultrasound images. The post-gadolinium images do not show abnormal contrast enhancement. The animals were maintained under general anesthesia throughout, then euthanized while still under anesthesia with an intravenous pentobarbital overdose.

The image spatial dimensions and sampling characteristics are presented in Table B of S1 File.

### Pulse motion measurement

Both of the imaging methods of this paper, optical imaging of human brain surface and cranial window ultrasound in a piglet, provide an opportunity for concurrent imaging of angiographic phenomena and brain motion. With the human brain surface optical data, the brain motion is measured on the visible wavelength recording whereas in the piglet cranial window ultrasound it is measured on the same same recording that shows the angiographic contrast passage. The two methods are otherwise identical and pulse brain motion is computed for the human and piglet data with the same source code. A region of interest (ROI) for motion tracking was drawn on an image of a video sequence, and the 2D translations of the ROI that maximize their correlation were taken to represent the motion.

**Fig 2:**
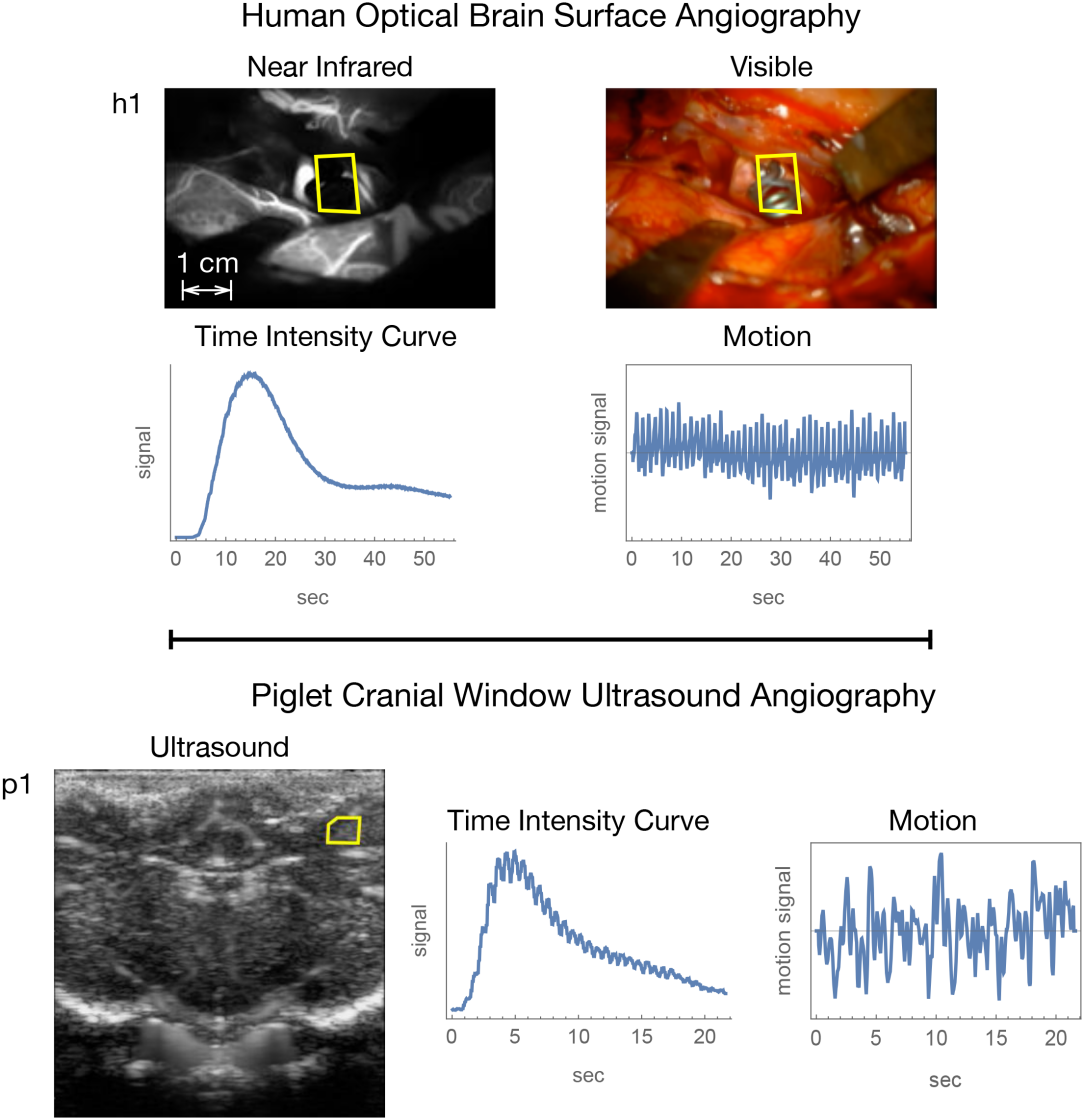
Representative snapshot images and temporal data summaries for human subject h1 and piglet subject p1. The yellow polygon in the images gives the region for motion tracking.

The regions selected for pulse motion tracking in human brain surface optical angiography are shown for h1 in Fig 2 and for h2-h4 in Fig D of S1 File. Freeze frame images of the ultrasound video sequences along with the regions selected for pulse motion tracking are shown for p1 in Fig 2 and for p2-p3 in Fig G of S1 File.

### Wavelet angiography

#### Time frequency resolution

Complex-valued wavelets have properties useful for mapping the temporal and spatial behavior of CF angiographic phenomena [24–26]. Unlike an unmodified Fourier transform, a wavelet transform can represent time varying CF phenomena. In addition, a complex number may be rendered conveniently as a pixel in a raster computer display by using polar coordinates in a brightness-hue color model. If *c* = *a* + *ib* represents a complex-valued CF datum in a spatiotemporal grid with real component *a* and imaginary component *b*, it has a polar representation {|*c*|, *ϕ*_*c*_} where magnitude $\left|c\right|=\sqrt{a^{2}+b^{2}}$ may be rendered in a pixel as brightness and *ϕ*_*c*_, the angle between the positive x-axis and the point {*a*, *b*}, as hue (Fig H of S1 File).

Whereas Fourier methods make reference to a signal’s frequency, by convention wavelet methods make reference to scale, *s*, which corresponds roughly to period (the reciprocal of frequency). Of particular importance to this study is the wavelet scale that corresponds to CF, termed *s*_♡_ in this paper. The value of *s*_♡_ is particular to the heart rate during a particular angiogram. Generally *s*_♡_ is larger for humans than for piglets because the piglet heart rate is about 3 times faster than the human heart rate.

The complex continuous wavelet transform of a signal *f*(*t*) is calculated by reference to a mother wavelet *ψ*, as given by the equation

$$\begin{array}{c} W\left(u,s\right)=\frac{\int_{-\infty }^{\infty }f\left(t\right)\psi^{*}(\frac{t-u}{s})\;dt}{\sqrt{s}}. \end{array}$$ (1)

where *s* is wavelet scale, as above, *u* is the wavelet translation parameter, and the superscript * represents complex conjugation [27]. The mother wavelet *ψ* controls the temporal and frequency resolution of the wavelet transform. The wavelet cine imaging methods developed in this paper make use of wavelets of a range of frequency and temporal resolution. A mother wavelet family that offers an explicit balancing of frequency and temporal resolution is the Gabor (variably termed the complex Morlet) wavelet *ψ*, given in principle by the equation

$$\begin{array}{c} \psi \left(t\right)=\frac{1}{\sqrt[{4}]{\pi }}e^{-\frac{t^{2}}{r^{2}}}e^{i\;t\;n\;} \end{array}$$ (2)

where the parameters *r* and *n* mediate the relation between the Gaussian $e^{-\frac{t^{2}}{r^{2}}}$, which shapes the temporal resolution, and the complex sinusoid *e*^*itn*^ = *cos*(*tn*) + *i sin*(*tn*), which gives the frequency specification [25, 26, 28–30]. The Gabor wavelet *ψ* family is used here to provide wavelet transforms of varying frequency and temporal resolution.

I employ over symbol notation in this paper to specify the frequency versus temporal resolution of a wavelet transform. A wavelet transform with unspecified resolution is denoted by the over bar symbol ^-^ (for example, ${\bar{c}}_{i,j}$ is a wavelet transform of unspecified resolution of *c*_*i*,*j*_, which represents the angiographic time intensity curve at the *i*, *j*^*th*^ pixel), a high temporal resolution wavelet transform is denoted by the over hat symbol ^ (as in ${\hat{c}}_{i,j}$), and a high frequency resolution wavelet transform is denoted by the over tilde symbol ^~^ (as in ${\tilde{c}}_{i,j}$). Uniformly spaced time sampling is implied and the subscript *t* is generally omitted. Lower case symbols are used to denote pixel-wise time signals, in which case the pixel indices *i*, *j* may be implied. Upper case symbols are used to denote frame wise signals. Table C of S1 File summarizes the mathematical notation used in this manuscript.

In this paper, filtering by CF wavelet scale *s*_♡_ at inverse wavelet transformation is implied, where “filtering” means nullification of all wavelet coefficients other than those for *s*_♡_ prior to inverse transformation.

The initial, naive, vascular PW cine imaging strategy in this paper was to reformat an angiogram from t frames of *n* × *m* pixels to an *n* × *m* array of signals each of length t, perform pixel-wise wavelet filtering for CF phenomena on each time signal, reformat back to t frames of *n* × *m* complex value data, and render each complex datum with the brightness-hue color model (Fig H of S1 File).

In the notation of this paper, the naive strategy was to compute ${\bar{c}}_{i,j}$ for the time signal of each *i*, *j*^*th*^ pixel, filter for *s*_♡_, inverse wavelet transform, and render. The Results section shows that this strategy proved unsuccessful, prompting the creation of numerically simulated angiographic data. The simulations, in turn, permitted the development of a reconstruction strategy based on the cross-correlation of wavelets of mixed high frequency and temporal resolution.

#### Computational implementation of Gabor complex-valued wavelets

The wavelet library employed here (Mathematica 11, Wolfram Research, Urbana, Illinois, USA) was subjected to acceptance testing as described in Section E-B of S1 File. This wavelet computational library provides a function, *GaborWavelet*[*n*], that reifies the Gabor wavelet *ψ* as described in Eq 2. The programmer chooses the value of the parameter *n* to control the comparative temporal and frequency resolution of the wavelet transformation at computation. The acceptance testing led to the selection of the particular *n* parameter values of 1 for high temporal resolution wavelet transformation and equivalently 6 or 12 for high frequency wavelet transformation.

#### Two-dimensional simulated optical angiography

The initial wavelet strategy for PW imaging proved unsatisfactory due to overwhelming artifact. The findings are shown in the Results section below. To develop a refined method of wavelet PW imaging, I developed a system for generating numerically simulated optical angiograms. The idea is to generate optical angiograms with known pulse motion and vascular pulsation to see if a given wavelet reconstruction method can recover the correct result.

A still NIR human image is obtained by averaging 300 NIR image frames of an example NIR video. To simulate tissue motion, the still image is computationally translated to and fro with bilinear 2-D interpolation according to the two-dimensional motions measured from the visible video. This is illustrated in exaggerated form by S40 Video. To simulate CF flow variation the intensity of each pixel is varied at two summed frequency components: the frequency of one event per sampling interval where the signal intensity is low at the start and end of the sampling interval and reaches a peak at the midpoint, to simulate bolus passage, and at CF. Pulse motion and flow variation are variably mixed into the same simulation. The video files for the simulated angiograms are supplied as S40 through S52 Videos as listed in Table E of S1 File.

#### One-dimensional simulated optical angiography

In an angiogram, a given pixel may be taken to represent a one-dimensional angiographic time intensity curve. To pursue wavelet reconstruction methods at the level of a pixel-wise time intensity curve, I generated simulated angiographic time intensity curves for analysis. These one-dimensional signals variably contain a synthetic bolus passage, CF variation, and random noise. They are subjected to wavelet transformation with wavelet *ψ*s of varying temporal and frequency resolution, and the result is compared to the known properties of the synthetic signal.

### Angiographic time of flight and arteriovenous pixel classification

ATOF is used to classify pixels as arterial versus venous. I evaluated several methods for estimating ATOF to a pixel including time to peak signal value, mean arrival time, and the phase of the frequency one Fourier component of the pixel’s circulatory time signal. The frequency one component of the Fourier transform has properties that are helpful in the modeling of a bolus passage. Like a bolus passage, it is a sinusoid event that occurs once per sampling interval. It has some advantages over other parameters such as the mean arrival time. For example, it filters out signals at other frequencies that do not correspond to the bolus passage, such as respiratory frequency and CF phenomena. Analysis with these methods of estimating ATOF reveals them to perform roughly equivalently with the human data but not with the piglet data. Testing shows the most robust results with the piglet data to be yielded by the phase of the Fourier frequency one component, so this method was used for both the human and piglet data.

The magnitude of the Fourier frequency one component relates to the areas under the time intensity curve, providing an analogy to the area under the curve in tracer kinetic methods [31, 32]. To enhance comparisons between subjects and species in these data, bolus passage time is normalized by heart rate such that for a given subject it ranges from 0 to the number of heart beats recorded in a sampling interval. Since the angiographic images are obtained at faster than cardiac rate, ATOF is measured here with fractional heart beat precision.

### Time-indexed phase histogram

The distribution of PW phase in a given time slice may be analyzed by a phase histogram. The comparative distributions of arterial and venous phase in a time slice may be analyzed by comparing phase histograms of each. The temporal progression of arterial and venous phase may be analyzed by constructing a phase histogram of each for each time slice followed by aligning these in sequence to produce a time-indexed phase histogram. A time-indexed phase histogram has three dimensions. The horizontal axis is time, expressed as heartbeats to facilitate comparisons between the human and piglet data. The vertical axis is PW phase, ranging as [−*π*, *π*]. The third dimension is relative histogram count, reflected in pixel brightness, with arterial phase counts in red and venous in blue. Fig R of S1 File illustrates the steps in generating a time-indexed phase histogram with arteriovenous classification.

## Results

The Methods section above describes the physical data acquisition and the baseline computational methods for PW imaging. Since the initial results contain dense artifact, the computational methods were refined to offer interpretable results. The initial results, the refinement of the computational methods, and the yielded results are described in this section.

### Angiographic and motion data

The figures in this document for the human and piglet data are based on subjects h1 and p1 (summarized in Fig 2). The figures for the other subjects are in S1 File. The video files that comprise this paper’s raw data and video renderings are provided as Supporting Information Videos S1 through S39 Videos as further described and listed in Table D of S1 File. The aneurysm location, heart rate, and signal to noise ratios (SNRs) for the CF component of the bolus passage and brain pulse motion are given in Tables A and B of S1 File for the human and piglet data respectively.

### Naive wavelet filter

**Fig 3:**
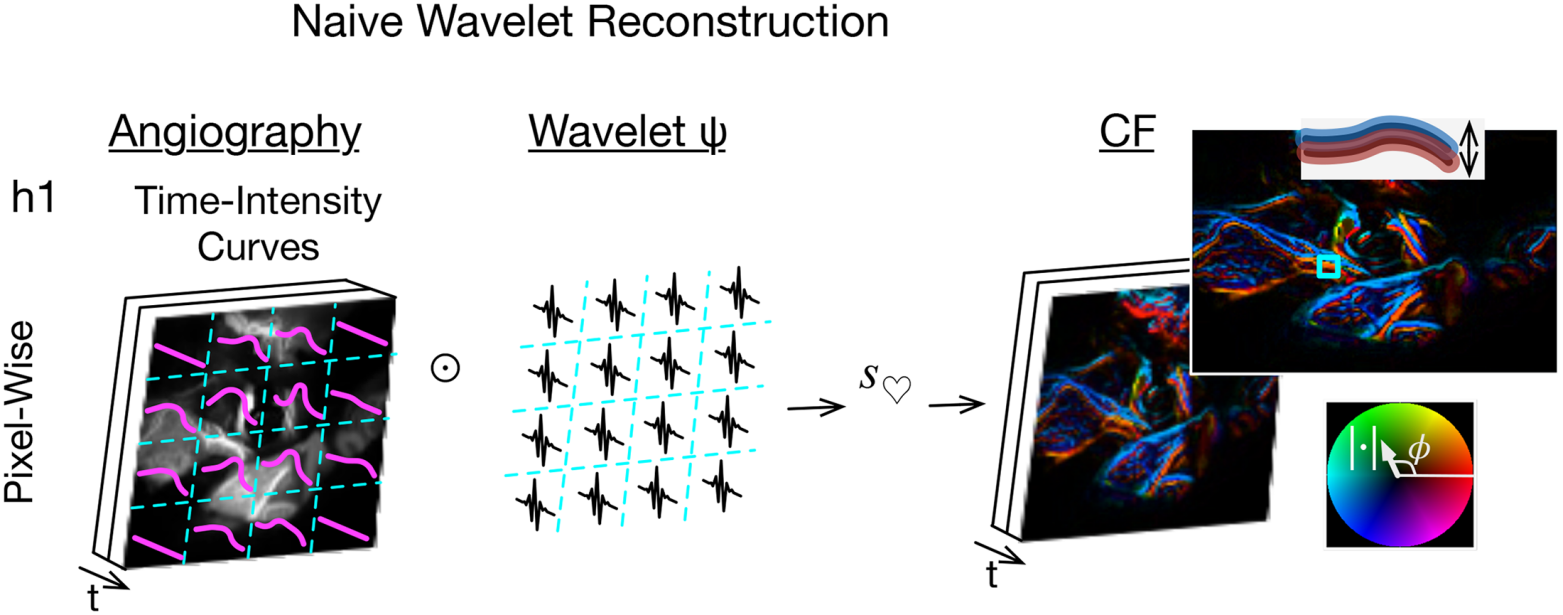
Naive wavelet angiography and motion alias. Apply wavelet transforms (⊙) to the pixel-wise time signals, filter for cardiac wavelet scale (*s*_♡_), and render by a brightness-hue color model that represents CF magnitude as brightness and phase as hue (right bottom inset). The double inset top right shows motion alias in the lengthwise bimodal phase.

The filter method proved unsatisfactory for the human optical data because the rendered images have excess artifact that appears as phase bimodality that is oriented longitudinally along vessels (Fig 3). The wavelet reconstructions offered by this method are attached as Supporting Information Video Files, with their file names given by the column headed by the symbol $\tilde{c}$ in Table D of S1 File. An explanation is that there is CF pulse tissue motion perpendicular to the course of the vessel that causes the vessel to shift side to side at CF. This causes contrast in the vessel to shift to and from adjacent regions perpendicular to the course of the vessel at CF, producing the longitudinal bimodality, a putative artifact termed here motion alias.

### Simulated optical angiography

**Fig 4:**
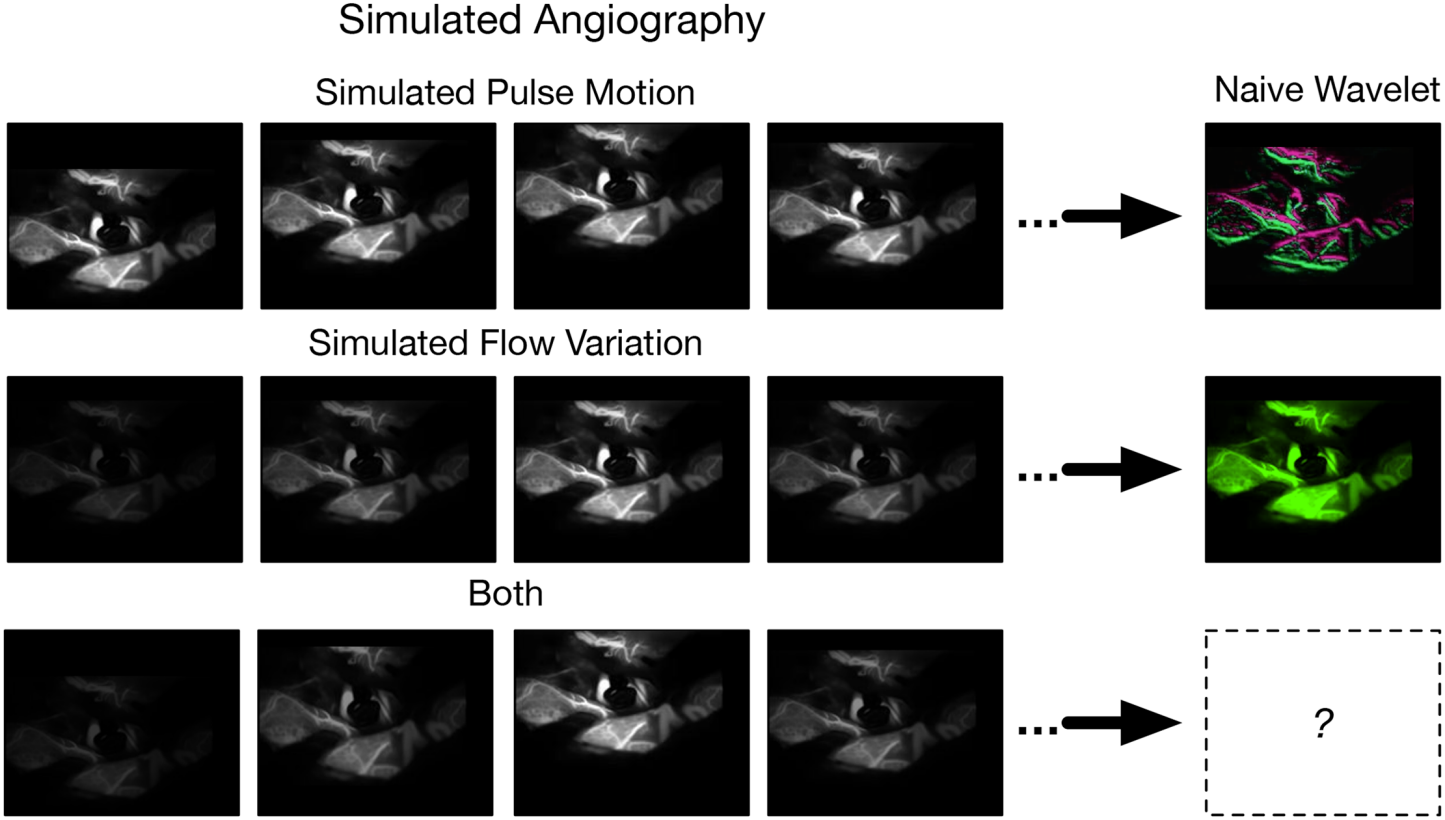
Optical angiogram simulation. The top row is simulated pulse motion. The bottom row is simulated intraluminal contrast variation. The right column shows an image after naive CF wavelet filtering. The simulated pulse motion and intraluminal contrast variation can be combined.

The simulated optical angiograms are filtered for CF angiographic phenomena using wavelet *ψ*s of varying frequency and temporal resolution. Some two-dimensional simulated optical angiograms and their wavelet reconstructions are attached as Supporting Information Video Files are provided as S40 through S52 Videos as listed in Table E of S1 File. Inspection of the results from the naive CF wavelet filter as applied to the simulated angiograms discloses that:

1. If there is CF pulse motion but no flow variation, then the result has motion alias, regardless of the wavelet *ψ* (first row of Fig 4).
2. If there is CF flow variation but no motion, then the result has no motion alias, regardless of the wavelet *ψ* (second row of Fig 4).
3. If there is both CF motion and flow variation, then there is a proportionate mixture of these where a high frequency resolution reconstruction does but high temporal resolution does not have motion alias.

Since the longitudinal bimodality can be reproduced in naive wavelet reconstructions of simulated angiograms with high proportions of pulse motion, the longitudinal bimodality of phase must reflect motion alias. Moreover, the results show that motion alias can be mitigated by filtering with a high temporal resolution *ψ*.

However, reliance on a high temporal resolution *ψ* may produce other facts due to the loss of frequency resolution. Testing against simulated one-dimensional data representative of a pixel-wise time signal shows that a naive CF wavelet filter based on a high temporal wavelet transform lets non-CF phenomena pass through, giving a separate type of artifact, frequency alias (Fig L of S1 File).

Further testing with one-dimensional simulated data shows that motion and frequency alias may jointly be mitigated by the cross-correlation of high temporal wavelet transforms of the pixel-wise time signals by a high frequency resolution wavelet transform of the frame wise time intensity curve (giving $\tilde{C}$, where the subscript *t* is implied). Please see Section E-C of S1 File particularly Fig M for details). The cross-correlation is performed by element wise multiplication after wavelet transformation [33–35].

In the notation of this paper, the proposed spatiotemporal CF filter by wavelet cross-correlation is then

$$\begin{array}{c} \hat{x}=\hat{c}\;{\tilde{C}}^{*}. \end{array}$$ (3)

**Fig 5:**
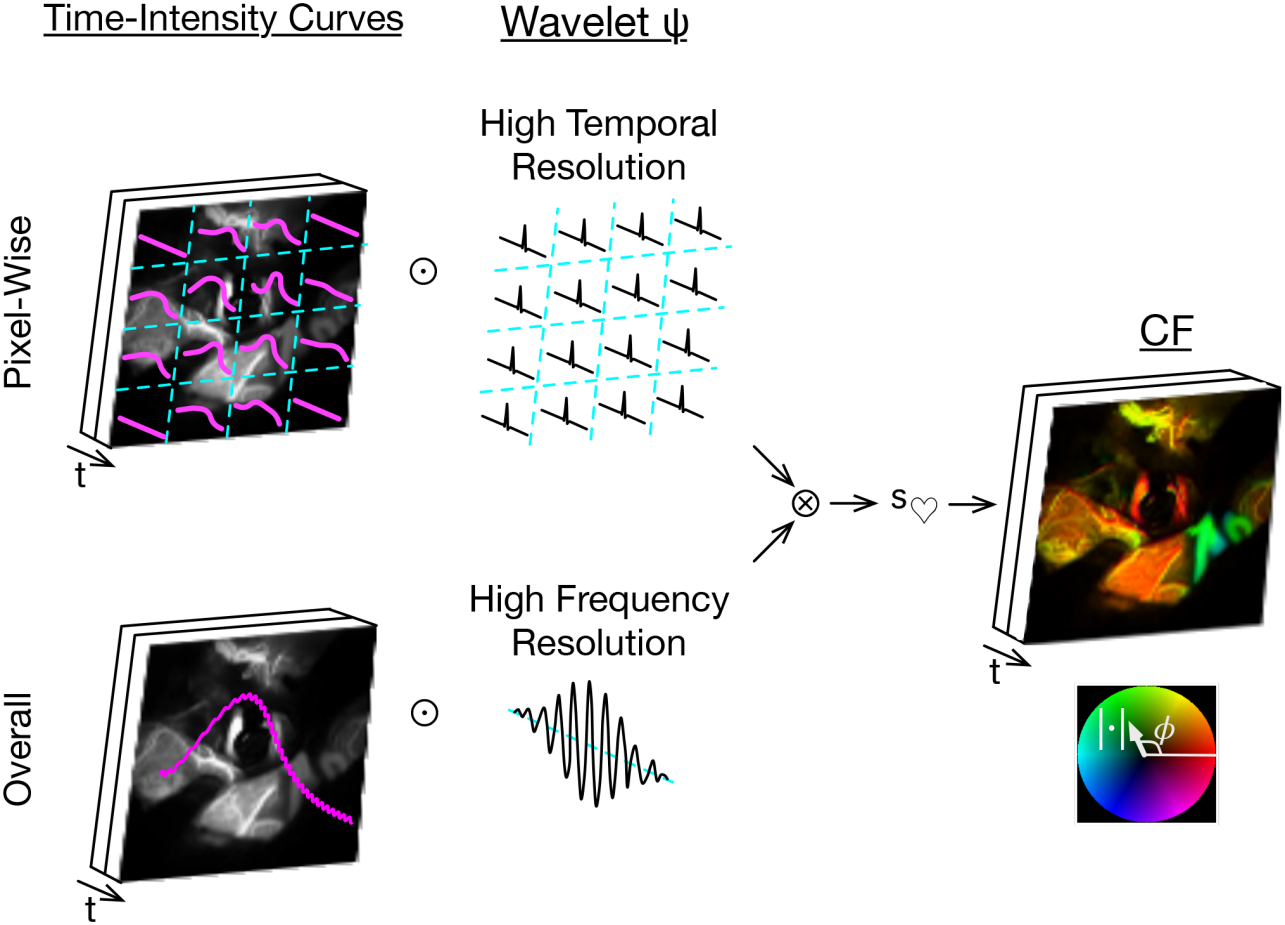
Cross-correlated wavelet angiography. Top left, apply high temporal resolution wavelet (⊙) transformation to the pixel-wise time intensity curves. Bottom left, apply high frequency resolution wavelet transformation to the overall time intensity curve. Right side, cross-correlate these pixel-wise (⊗), filter for cardiac wavelet scale (*s*_♡_), inverse wavelet transform, then cine render.

The time domain VPW cine images are obtained by inverse wavelet transformation of $\hat{x}$ with filtering by *s*_♡_ (Fig 5, for details see the Materials and methods section).

#### Mitigation of artifact summary

The two-dimensional and one-dimensional optical angiograms simulations suggest the following strategy for mitigating artifact at wavelet angiography:

1. Employ a high temporal resolution wavelet *ψ* for the pixel-wise circulatory signal transforms ${\hat{c}}_{i,j}$ to mitigate motion alias.
2. Apply a high frequency resolution wavelet *ψ* transform to the overall circulatory signal normalized as $\frac{\tilde{C}}{|\tilde{C}|}$.
3. Perform pixel-wise cross-correlation as ${\hat{x}}_{t,i,j}={\hat{c}}_{t,i,j}\frac{{\tilde{C}}_{t}^{*}}{|{\tilde{C}}_{t}|}$ to restore the loss of frequency resolution from the pixel-wise high temporal resolution *ψ* transform, with a possible second order correction to counter residual low frequency alias from the bolus passage.
4. Apply mean signal norming as point-wise multiplication along t of *x*_*i*,*j*_ by *C*_*t*_.

Please see Sections E-C and E-D of S1 File for further details on these artifacts and their mitigation.

### Reference to pulse motion

Having produced a computational wavelet system for cine imaging vascular PW that mitigates motion and frequency alias, we turn attention to developing a way of applying motion referencing to cine images of vascular PWs. The definition of $\hat{x}$ may be adjusted to include reference to CF pulse motion by adjusting with complex-valued arithmetic the high frequency correland $\tilde{C}$ in Eq 4 by the phase of pulse motion, $\tilde{M}$, to give $\tilde{C}{\tilde{M}}^{*}$. Substituting $\tilde{C}{\tilde{M}}^{*}$ for $\tilde{C}$ in Eq 3 gives

$$\begin{array}{c} \hat{y}=\hat{c}{\left(\tilde{C}{\tilde{M}}^{*}\right)}^{*}=\hat{c}\;{\tilde{C}}^{*}\tilde{M}. \end{array}$$ (4)

The motion-referenced spatiotemporal decompositions given by $\hat{y}$ may be obtained as above by inverse wavelet transformation with filtering by *s*_♡_ and rendering as video files (see the file names in the last column of Table D of S1 File). Perusal of these video files shows the motion-referenced vascular PW cine images to look similar to the non-motion-referenced ones except that there is creative constancy of phase (rendered as color) across each heart beat. Time-indexed phase histograms of motion-referenced phase are constructed as for the time-indexed phase histograms above.

### Human and piglet vascular pulse wave imaging

Having refined the wavelet methods, we return to their application to the human and piglet data.

**Fig 6:**
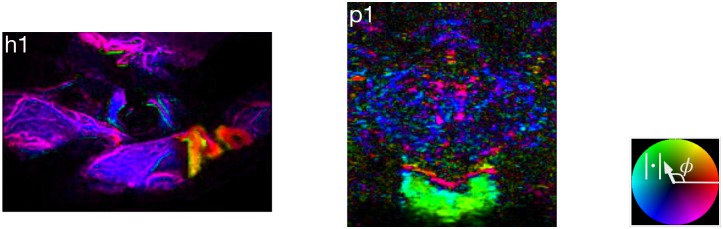
Wavelet angiography snapshots.

The vascular PW cine images may be rendered with the brightness-hue model as video files for inspection. The video files have three dimensions, two spatial and one temporal. The raw data video files and their wavelet PW reconstructions are described in Table D of S1 File and are provided as Supporting Information Videos S1 through S39 Videos. For representation on a printed page a two dimensional spatial snapshot may be selected from a given time coordinate. Selected snapshots of $\hat{x}$ for h1 and p1 are presented in Fig 6 and for the other subjects in Fig O of S1 File.

### Angiographic time of flight and arteriovenous pixel classification

**Fig 7:**
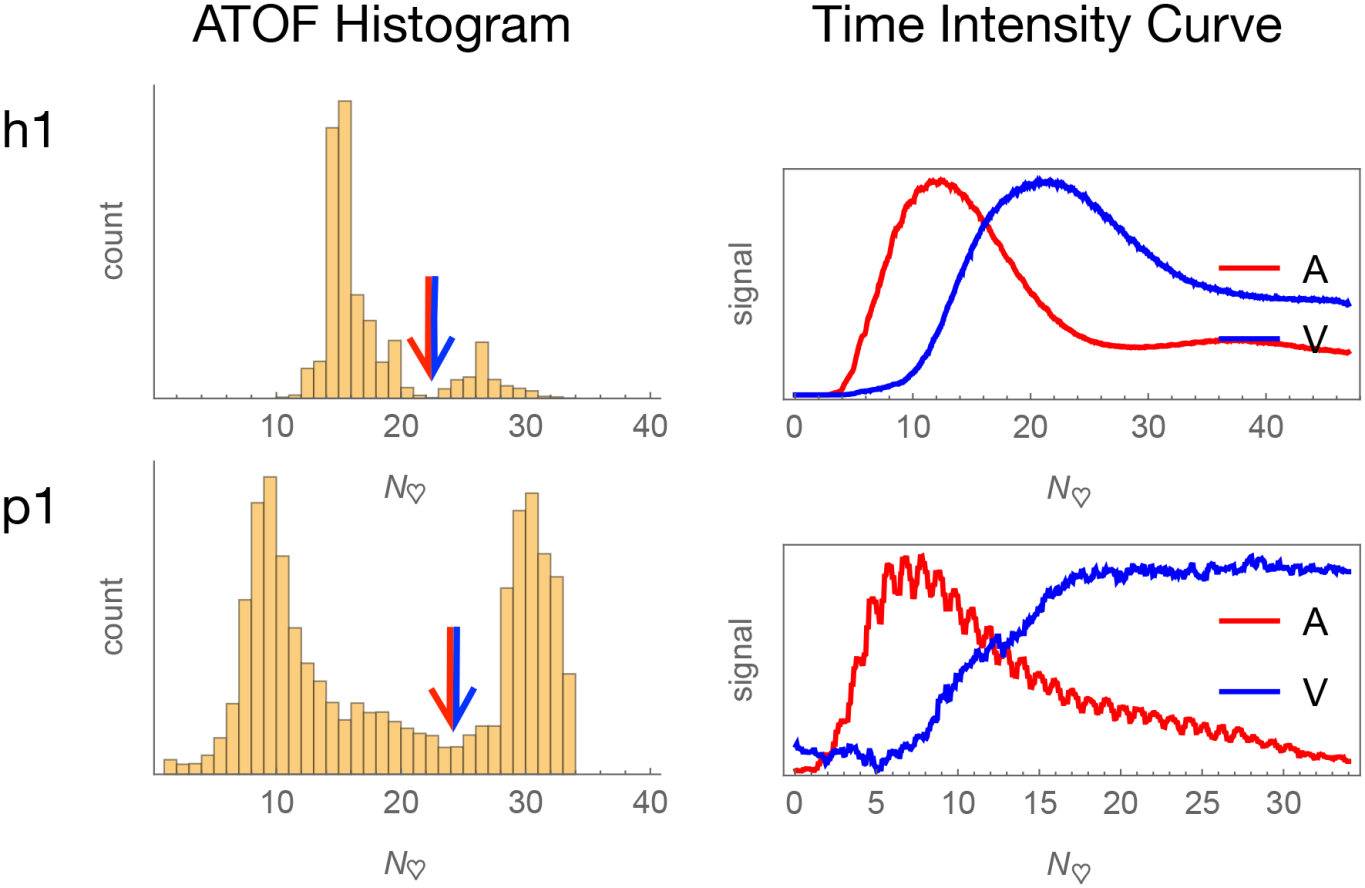
Arteriovenous classification. Arterial and venous pixels are separated according to angiographic time of flight (ATOF). The derived time intensity curves are respectively consistent with predominantly arterial and venous temporal behavior.

Histograms of ATOF generally approximate a bimodal shape (Fig 7 and Fig P of S1 File). There is extended venous stasis and evidence of angiographic recirculation in some of the subjects that impairs the estimation of the mean angiographic transit time, but inspection of the ATOF histograms suggests it to be in the range of 10 to 40 heartbeats across the subjects. For each subject, the ATOF value of the deepest valley was selected as the arterial versus venous classification threshold. For the humans, those vessels classified as arterial versus venous corresponds to the impression based on visual inspection of the actual surgical field and the visible video. For the piglets, the classification of major arteries and veins corresponds roughly to a piglet atlas (Fig F of S1 File). There must be some statistical error in the binary classification of pixels as arterial versus venous. Nonetheless, the time intensity curves drawn from pixels classified as arterial and venous have shapes consistent respectively with the arterial and venous circulations because the time intensity curves drawn from pixels classified as arterial peak earlier than the time intensity curves from pixels classified as venous.

### Arterial and venous time-indexed phase histograms

**Fig 8:**
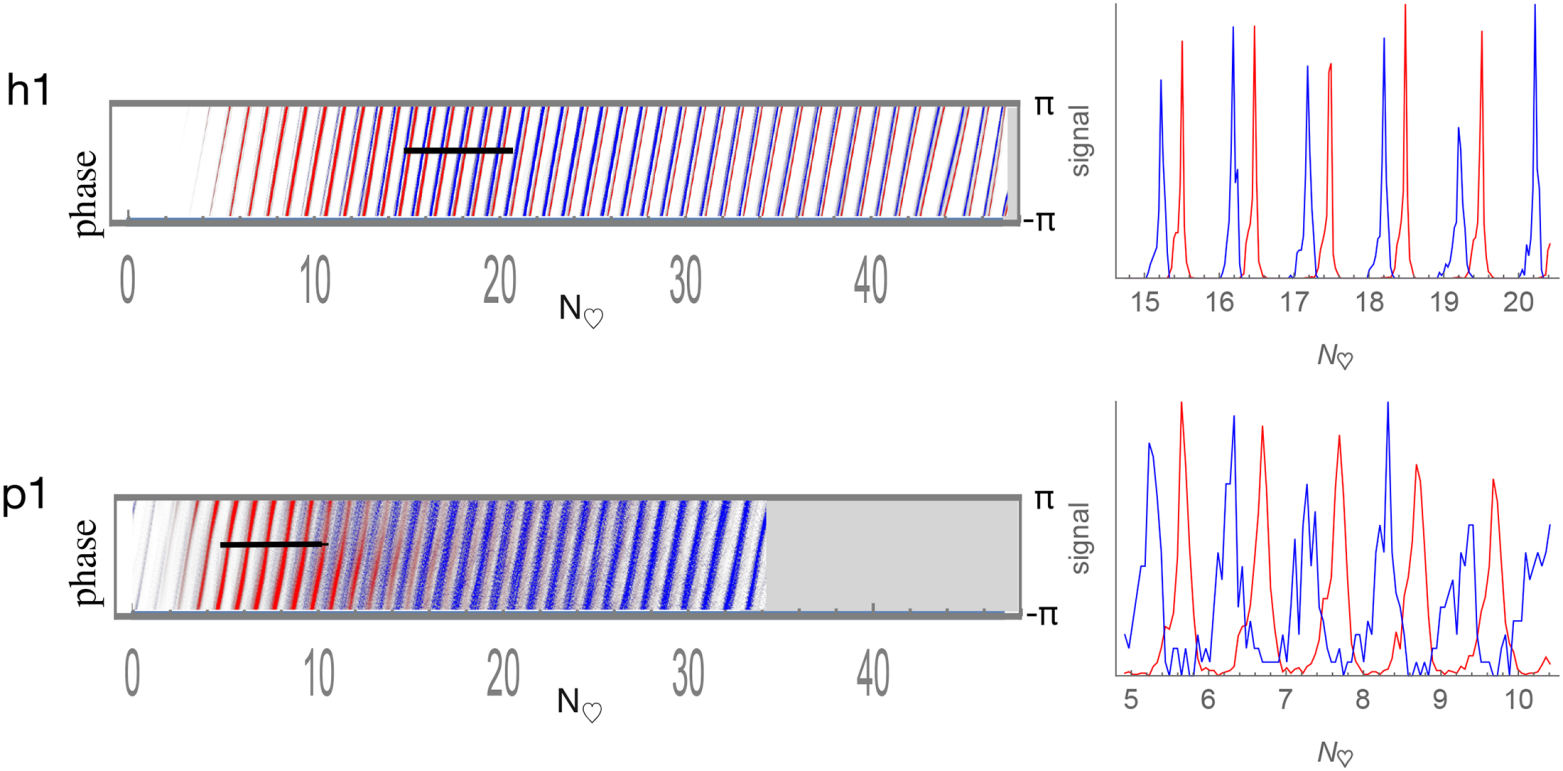
Arterial and venous time-indexed phase histograms with line scans. Pixels classified by ATOF as arterial are in red and those as venous in blue. Relative histogram count is represented as brightness. The horizontal axis is time is depicted in heartbeats to facilitate human-piglet comparison. The vertical axis is phase ranging as [−*π*, *π*]. There is a consistent arteriovenous phase difference across the bolus passage.

The time-indexed phase histograms for subjects h1 and p1 are shown in Fig 8 and for the other subjects in Fig S of S1 File. Consistent with the ATOF criteria employed to classify pixels as arterial versus venous, red (arterial) comparatively predominates on the left early in the bolus passage and blue (venous) on the right.

Visual inspection of the merged arterial and venous time-indexed phase histograms appears to show the presence of a consistent arterial versus venous phase difference across the bolus passage. Line scans are drawn through each of the time-indexed-phase histograms to depict further detail on arterial and venous phase (second column of both Fig 8 and Fig S of S1 File). These data may be interpreted as showing the presence of resolvable arterial and venous pulsations in both human and piglet brain.

### Arterial and venous phase histograms after reference to brain pulse motion

**Fig 9:**
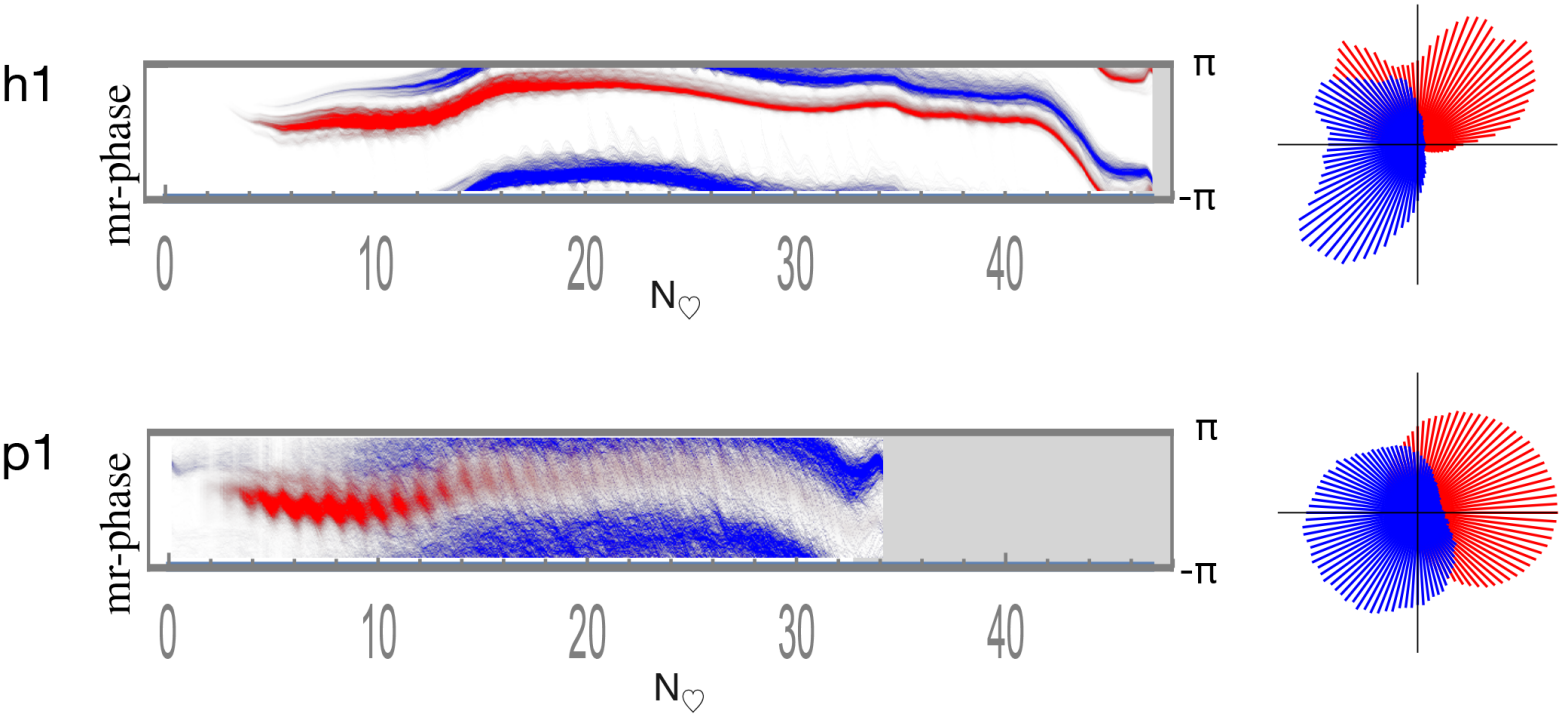
Arterial and venous motion-referenced phase histograms. Pixels classified by ATOF as arterial are in red and those as venous in blue. Relative histogram count is represented as brightness. The horizontal axis is time is depicted in heartbeats to facilitate human-piglet comparison. The vertical axis is phase ranging as [−*π*, *π*]. Arterial versus venous phase appears approximately fixed in difference across the entire bolus passage. The circular phase histograms on the right are calculated by summing the time-indexed phase histograms on the left across time.

The phase histograms may be computed from the motion-referenced wavelet angiograms to show the relationship between angiographic PW phase and pulse motion phase. Fig 9 shows the motion-referenced time-indexed phase histograms for subjects h1 and p1. The other subjects are shown in Fig T of S1 File. The arterial and venous phases maintain a relatively constant phase difference across the bolus passage consistent with an arteriovenous phase lock. When the motion-referenced phases are summed across the time of the bolus passage and represented as circular histograms, there remains an overall arteriovenous PW phase difference (right column of both Fig 9 and Fig T of S1 File).

## Discussion

This paper reports exploratory vascular PW cine imaging by a wavelet angiography method capable of resolving individual PWs, and finds arterial and venous PWs to have separate phase relations to each other and to brain pulse motion in both humans and piglets (Figs 8 and 9). The arteriovenous phase lock is consistent with the hypothesis that there is information exchange between the arterial and venous components of cerebral circulation. This phase lock may be an experimental signature of a coordinating mechanism whereby an arriving stroke volume of arterial blood matches to an equivalent ejection of venous blood.

These exploratory data furthermore suggest the presence of a trilateral phase relationship relationship between the phases of arterial PWs, venous PWs, and brain pulse motion. A rudimentary interpretation of these data is that arterial wall expansion displaces extravascular tissues to produce venous wall compression by local conservation of mass. Even such a simple model implies complex topological arrangements in tissue between arterial and venous vasculature and places constraints on the biophysical properties of the intervening brain tissue [36]. The presence of these implied properties may be subject to experimental testing. These data permit no comment on whether the brain has an active regulatory system with PW sensors integrated to vascular effectors that can modulate spatiotemporal PW phenomena. The potential role of alterations in biomechanical tissue properties on arteriovenous PW interactions is a topic for further research.

I do not report data outside of the brain but it is possible that parallel arteriovenous information exchange occurs throughout the entire central nervous system including within the intradural spinal component [37].

### Limitations

Of the four human subject set aside for wavelet analysis, the features of their optical angiograms that make them suitable for wavelet analysis (no motion of the microscope, no manipulation of the vessels, and inclusion of the venous phase of the angiogram) seem unlikely to have influence that distribution of vascular PWs and of brain pulse motion. Therefore I assess as unlikely that the core findings reported here are unduly influenced by a selection factor.

The geometry of human brain surface optical angiography proves well suited to the detection of motion alias because the exposed brain surface vessels are perpendicular to the line of sight of the operating microscope. The same surface geometry simplifies the numerical simulation of angiography. The angiographic simulation could be improved by the incorporation of other signal sources such as respiratory frequency phenomena.

By contrast to optical brain surface angiography, the cross-sectional geometry of the piglet cranial window ultrasound angiography method does not lend itself as easily neither to the detection of angiographic motion alias nor to the generation of simulated angiography, since such would require perhaps a three dimensional simulation. Nonetheless, the measurability of pulse motion in the raw cine data is consistent with the presence of motion alias and justifies the use of the cross-correlated wavelet method.

Neither of these two angiographic methods captures three dimensional spatial data. The suggestion here of arterial and venous PW complementarity is based on the observation of arteriovenous phase locking. Were a three dimensional wavelet angiography method to be employed, one would experimentally predict the the presence of CF oscillating arterial and venous blood volumes that sum to an approximate constant in the central nervous system throughout the cardiac cycle [38].

This cross-correlated wavelet method attenuates both motion and frequency alias sufficient for testing the hypothesis of this paper, but the region of interest for pulse motion tracking is selected opportunistically by visual inspection. The location of the region of motion tracking interest influences the individual phase relationships between pulse motion and arterial and venous PWs but it does not impact the measured phase differences between arterial and venous PWs.

Since the brain surface optical technique is performed at open cerebrovascular craniotomy, there is no intact cranial vault to constrain brain pulse motions. This factor limits the generalizability of these human data since pulse motion may be of lower magnitude when the skull is intact. If further research were to establish that there is less pulse brain motion in human high speed angiographic data acquired with a closed cranium, it might be feasible to mitigate motion alias with a wavelet *ψ* of less high temporal resolution than employed here, thereby mitigating the frequency alias that comes with the use of an extreme high temporal resolution wavelet *ψ*.

### The ICP waveform

The management of ICP via implanted devices plays a role in the care of patients with closed head injury, spontaneous intracerebral hemorrhage, and cerebral edema [14, 39]. There is CF variation to ICP but the basis of the ICP waveform remains unknown [40, 41]. The addition of a method such as reported here for vascular PW cine imaging may enable an experimental strategy for reconstructing the causal chain that produces the ICP waveform.

### The brain ventricles

The relationships between brain pulse motion and arterial and venous PWs reported here raise the question of whether an arterial PW may cause coordinated pulse motions of brain ventricle walls [42]. The low viscosity and lack of internal structure to CSF may enable the ventricles to provide a low friction relay medium for PW coupling, possibly extending the spatiotemporal window for effective coupling. This formulation experimentally predicts an association between vascular PWs about the surfaces of the ventricles, ventricle walls motions, and adjacent internal CSF pressure variations.

Selected human patients with hydrocephalus who undergo attempted endoscopic treatment offer a brief opportunity for the internal direct observation of ventricle wall motions and adjacent CSF pressure variations [43]. In a related article, my coauthors and I report direct intraoperative observations from within the lateral and third ventricles of three such human subjects and we compare the measurements to the distribution in piglets of vascular PWs over the ventricular surfaces [3].

These and further studies may shed light on whether the ventricles serve to shape a low viscosity medium, CSF, so as to enable vascular PW coupling across remote vascular distributions. The ventricles remain the largest structure in the human body without established primary purpose, even though their existence has been known at least since 350 BCE when Aristotle observed of the brain “in the great majority of animals it has a small hollow in its centre” [44].

## Supporting information

S1 Video Visible brain surface video for human subject h1. (MOV)

S2 Video Optical brain surface angiography video for human subject h1. (MOV)

S3 Video High temporal resolution CF wavelet filter of S2 Video. (MOV)

S4 Video High frequency resolution CF wavelet filter of S2 Video. (MOV)

S5 Video Cross-correlated wavelet CF wavelet filter of S2 Video. (MOV)

S6 Video Motion-referenced cross-correlated wavelet CF wavelet filter of S2 Video. (MOV)

S7 Video Visible brain surface video for human subject h2. (MOV)

S8 Video Optical brain surface angiography video for human subject h2. (MOV)

S9 Video High temporal resolution CF wavelet filter of S8 Video. (MOV)

S10 Video High frequency resolution CF wavelet filter of S8 Video. (MOV)

S11 Video Cross-correlated wavelet CF wavelet filter of S8 Video. (MOV)

S12 Video Motion-referenced cross-correlated wavelet CF wavelet filter of S8 Video. (MOV)

S13 Video Visible brain surface video for human subject h3. (MOV)

S14 Video Optical brain surface angiography video for human subject h3. (MOV)

S15 Video High temporal resolution CF wavelet filter of S14 Video. (MOV)

S16 Video High frequency resolution CF wavelet filter of S14 Video. (MOV)

S17 Video Cross-correlated wavelet CF wavelet filter of S14 Video. (MOV)

S18 Video Motion-referenced cross-correlated wavelet CF wavelet filter of S14 Video. (MOV)

S19 Video Visible brain surface video for human subject h4. (MOV)

S20 Video Optical brain surface angiography video for human subject h4. (MOV)

S21 Video High temporal resolution CF wavelet filter of S20 Video. (MOV)

S22 Video High frequency resolution CF wavelet filter of S20 Video. (MOV)

S23 Video Cross-correlated wavelet CF wavelet filter of S20 Video. (MOV)

S24 Video Motion-referenced cross-correlated wavelet CF wavelet filter of S20 Video. (MOV)

S25 Video Cranial window ultrasound angiography video for piglet subject p1. (MOV)

S26 Video High temporal resolution CF wavelet filter of S25 Video. (MOV)

S27 Video High frequency resolution CF wavelet filter of S25 Video. (MOV)

S28 Video Cross-correlated wavelet CF wavelet filter of S25 Video. (MOV)

S29 Video Motion-referenced cross-correlated wavelet CF wavelet filter of S25 Video. (MOV)

S30 Video Cranial window ultrasound angiography video for piglet subject p2. (MOV)

S31 Video High temporal resolution CF wavelet filter of S30 Video. (MOV)

S32 Video High frequency resolution CF wavelet filter of S30 Video. (MOV)

S33 Video Cross-correlated wavelet CF wavelet filter of S30 Video. (MOV)

S34 Video Motion-referenced cross-correlated wavelet CF wavelet filter of S30 Video. (MOV)

S35 Video Cranial window ultrasound angiography video for piglet subject p3. (MOV)

S36 Video High temporal resolution CF wavelet filter of S35 Video. (MOV)

S37 Video High frequency resolution CF wavelet filter of S35 Video. (MOV)

S38 Video Cross-correlated wavelet CF wavelet filter of S35 Video. (MOV)

S39 Video Motion-referenced cross-correlated wavelet CF wavelet filter of S35 Video. (MOV)

S40 Video Simulated human brain surface optical angiogram with exaggerated pulse motion. (MOV)

S41 Video Simulated human brain surface optical angiogram with pulse motion. (MOV)

S42 Video High temporal resolution CF wavelet filter of simulated human brain surface optical angiogram with pulse motion. (MOV)

S43 Video High frequency resolution CF wavelet filter of simulated human brain surface optical angiogram with pulse motion. (MOV)

S44 Video Cross-correlated CF wavelet filter of simulated human brain surface optical angiogram with pulse motion. (MOV)

S45 Video Simulated human brain surface optical angiogram with pulse flow. (MOV)

S46 Video High temporal resolution CF wavelet filter of simulated human brain surface optical angiogram with pulse flow. (MOV)

S47 Video High frequency resolution CF wavelet filter of simulated human brain surface optical angiogram with pulse flow. (MOV)

S48 Video Cross-correlated CF wavelet filter of simulated human brain surface optical angiogram with pulse flow. (MOV)

S49 Video Simulated human brain surface optical angiogram with both pulse motion and pulse flow. (MOV)

S50 Video High temporal resolution CF wavelet filter of simulated human brain surface optical angiogram with both pulse motion and pulse flow. (MOV)

S51 Video High frequency resolution CF wavelet filter of simulated human brain surface optical angiogram with both pulse motion and pulse flow. (MOV)

S52 Video Cross-correlated CF wavelet filter of simulated human brain surface optical angiogram with both pulse motion and pulse flow. (MOV)

S53 Video Visible-NIR video alignment. Demo of graphical user interface program for synchronizing human visible and NIR videos.
(MP4)

S1 File Supporting information on methods and results for human and piglet wavelet angiography, mathematical methods and numerical angiography simulation. Contains supporting text, figures, and tables.
(PDF)
