# Supplementary material for: Wavelet brain angiography suggests arteriovenous pulse wave phase locking
Source: PLoS One. 2017 Nov 15;12(11):e0187014. doi: 10.1371/journal.pone.0187014 (PMC5687712; doi:10.1371/journal.pone.0187014)
Supplement: S1 File — Contains supporting text, figures, and tables. (PDF) [file pone.0187014.s054.pdf]


Supporting Information

William E. Butler

A Human Brain Surface Optical Angiography During Cerebrovascular Surgery

A Intraoperative Data Acquisition

The human subject portion of this paper represents a post-hoc analysis after discharge from the hospital of intraoperative video footage routinely recorded in the course of clinical care. No aspect of this study altered in any way the care of these patients. Craniotomy for microsurgical clipping was selected as the treatment strategy instead of endovascular aneurysm coiling based on aneurysm location and configuration [1]. Images from the preoperative head computed tomography angiogram depict the aneurysm and parents vessels for surgical planning (Fig A). For the angiography, the focal plane of the microscope is set to 300 mm, the zoom to 5x, and the illumination bulb to 50% power. The wavelength spectrum of the illumination bulb includes the 780 nm range of ICG excitation

B Preparation of Human Videos for Numerical Analysis

The two captured video files, one visible and one near infrared (NIR), are preprocessed for computational analysis. The command line program ffmpeg is used to convert the files into .mov format (version 2.1.5) [2]. During the conversion, the video files are reduced 1/2 size in the horizontal and vertical dimensions by linear sampling to enhance interactivity in the analysis. An analysis of visible video footage of a ruler determines that the 720 by 480 pixels of each video frame correspond to 53 pixels per centimeter horizontally and 48 pixels per centimeter vertically. This is the basis for the 1 cm markers in Fig D here and Figs 1 and 2 of the main document.

The videos are then imported in to the computational analysis environment Mathematica version 10 (Wolfram Research, Champagne, Illinois) for analysis. Initial analysis showed the earlier parts of the NIR video file with early dim signal to have greater amplification than the later brighter signal portions, consistent with variable amplification. I wrote a semi-automated routine to identify and reverse the variable amplification (Fig B).

Visual inspection the visible and NIR angiogram video files recored by the operating microscope reveals that they are not in synchrony when downloaded from the microscope, but instead are temporally misaligned by up to 11 frames (the image frequency is 30 Hz). Their timings must be adjusted to enable the analysis of motion and flow. At the end of the indocyanine green (ICG) run, the surgeon halts the recording by pressing a button on the microscope handle. Given that the microscope magnification is at 5x, the button press causes a notable simultaneous displacement in both the visible and NIR videos that can be used to synchronize them. I wrote a graphical user interface program to manipulate the temporal alignment of the visible and NIR video footage by reference to the sharp field of view displacement cause by the button press, and estimate that the videos are aligned thereby to within about 1 frame

(Fig C) . A demonstration of the use of this program video containing annotations in purple and edited for clarity of the use of this program is supplied as S53Video.

Summary data for the human cerebral aneurysms and their post-clipping optical angiograms are presented in Table A.

Snapshot visible and NIR images and temporal data summaries for subjects h2-4 are presented in Fig D.

B Piglet Cranial Window Ultrasound Angiography

A Perflutren Phantom Calibration

Perflutren (Definity, Lantheus Medical Imaging, North Billerica, Massachusetts) is an ultrasound contrast agent that mimics red blood cell rheology, is hemodynamically inert at diagnostic ultrasound frequency and intensity ranges [3,4], and enables quantitative perfusion imaging [5–12]. Evolving methods ultrasound imaging methods with increased sensitivity and speed appear likely to permit a fidelity and sampling rates beyond those employed here [13,14].

Imaging of the phantom and the piglets was at 8.3 MHz using a linear array probe (MySonos 201, Medison) with ultrasound gain dials (overall, near-field, and far-field) were set to 32 on the device. The analog images produced by the device were digitized (DT3162 Variable-Scan Monochrome Frame Grabber, Data Translation, Marlboro, Massachusetts, for early experiments, and Dazzle DVBridge, Pinnacle Systems, Mountain View, CA, for the latter experiments; I verified in vitro that both systems provided equivalent digitized values for the same phantom) and stored as cine image sequences in QuickTime format [2]. The cine images were then imported into the Mathematica environment for analysis by custom programs.

A 12-well tissue culture plate with a latex window was imaged with perflutren microspheres in different wells at concentrations of 10, 5, 1, 0.1 and 0.01 million microspheres per mL for 16 minutes. This method did not yield a monotonic concentration-to-signal relationship, as microspheres tend to rise to the surface in a stagnant system, preventing uniform mixing. Therefore, to simulate active mixing more typical of a physiologic system, I built closed-loop and open-loop phantoms. In vitro phantoms for assessing quantitative ultrasound have been employed by others [15].

The closed-loop phantom is designed to simulate steady-state flow. Polyvinyl tubing of volume 30 mL was attached to a peristaltic pump programmed to 100 mL per minute, providing a circular flow rate similar to that observed in mammalian arterial circulation. Images were obtained as the perflutren microsphere concentration was systematically varied (0, 1, 2.5, 5, 7.5 and 10 million microspheres per mL) in the flowing circuit.

The open-loop phantom is designed to simulate bolus passage. A syringe with a known quantity of perflutren was connected to an open segment of polyvinyl tubing with flowing saline solution. I injected the bolus of perflutren, imaged its passage with cine ultrasound, and recorded the total ultrasound signal intensity obtained. Fig E illustrates the relationship obtained by plotting the signal data from the closed-and open-loop phantoms, after background-subtraction. Both data sets show an approximate linear relationship between contrast concentration and signal intensity, suggesting that tracer kinetic methods are applicable to the ultrasound signals obtained from perflutren bolus passage in the piglet model described here [16].

B Preparation of Ultrasound Angiography for Analysis

Prior to computational analysis, for each cine ultrasound sequence a polygon was drawn on a representative ultrasound image to separate the intracranial region from the

remainder of the anatomy, as illustrated in Fig F. Based on this boundary, bony skull base structures and extracranial vessels were excluded from all analysis. To capture a motion signal, the ultrasound video was inspected, and a site of motion independent of the passage of contrast was identified. In two animals, this site was at the inferior border of the cranial cavity, and in one it was located peripherally in a fissure. In one animal, a site with consistent motion could not be identified, so it is excluded from further analysis because its vascular pulsations could hence be referenced to pulse motion. A polygon was drawn on each motion site, and a motion signal was measured using the same algorithm and software code as applied to the intraoperative human optical data. The coronal images, motion region of interests, and angiographic time intensity curves are shown for each of the subjects in Fig G here and Fig 2 of the main document.

Summary data for the cranial window ultrasound angiograms are presented in Table B;

Snapshot cranial window ultrasound angiogram images and temporal data summaries for subjects p2-3 are presented in Fig G.

C Pulse Motion Measurement

The same method and the same computer source code is employed to measure cardiac frequency (CF) pulse brain motion for the human brain surface optical and piglet cranial window ultrasound data. An angiogram video is inspected for an object or region with particularly clear pulse motion. Its spatial boundaries are marked by a polygon drawn in a graphical user interface on a still image from the video sequence. The pixels within the polygon define the region of interest (ROI) for motion tracking.

To reduce motion noise, the video frames are averaged with each frame replaced by the mean of the 5 nearest frames. The spatial displacement is calculated between each such frame pair 6 frames apart. This is selected instead of adjacent frames because too little motion generally occurred between adjacent frames to be consistently detectable. The policies of local frame averaging and of measuring motion across a frame interval of 5 were found by trial and error generally to yield satisfactory signal to noise ratios (SNRs) of the motion signal. The method of approximating CF SNR is described below in Section D.

For each such frame pair, the ROI in the second frame is displaced between -5 to +5 pixels both horizontally and vertically and each such displaced version is paired with the same first frame, giving $11 \times 11 = 121$ total combinations of ROI pairs. The pixel-wise correlation between the ROI of the first frame and each of the 121 displaced ROIs of the second frame are computed, giving a matrix of 121 correlations. The center of mass of these 121 correlations is taken to represent the net displacement vector of the ROI between the two frames. This displacement vector is then calculated for all successive frame pairs, giving $n-5$ displacement vectors for n frames, to which is prepended 2 copies and appended 3 copies of the null displacement vector $\{0., 0.\}$ (Fig I).

For convenience, the displacement vector is then reduced in dimensionality from a sequence of two-dimensional displacements to a one dimensional motion signal as follows. The two-dimensional displacements are plotted and treated as a scattergram. The covariance matrix of the scattergram is computed and subjected to eigen-decomposition. Then each individual two-dimensional displacement is projected onto the axis of the major eigenvector of the covariance matrix. This gives a sequence of scalar displacements that termed here the motion signal, M (indexing by uniformly sampled time intervals is implied). The SNRs yielded by these methods are shown for the human data in Table A and for the piglet data in Table B.

D Cardiac Frequency Signal To Noise Ratio

I employ Fourier methods to look for the presence of CF signal relative to noise before analyzing the time-resolved CF signal by wavelet methods. The Fourier analysis of SNR is based on dividing a narrow band signal of interest (CF in this instance) by a broadband background [17]. However in these data there are other signals in the broadband background that should not be counted as noise. These include the respiratory frequency signal and the very low frequency bolus passage signal. I exclude these from the broadband background in generating the denominator for SNR calculations. Instead the SNR is estimated here as the height of the Fourier spectral peak for CF divided by the mean height of 5 peaks starting about 10 steps away in the higher frequency direction. This appears to serve as a suitable broadband denominator in all signal data analyzed here, including angiographic and pulse motion data for both humans and piglets. The results, presented in Tables A and B, should be taken as mere approximations but they appear to support the presence in all of the data of a CF signal appropriate for further analysis.

E Wavelet Angiography Computational Methods

A Gabor Complex-Valued Wavelets

For a complex-valued CF datum $c = a + ib$ in a spatiotemporal grid, its magnitude $|c|$ is rendered in a pixel as brightness and its phase ϕ_c as hue (Fig H).

The complex continuous wavelet transform of $f(t)$ is given by the equation

$$W(u, s) = \frac{\int_{-\infty}^{\infty} f(t) \psi^* \left(\frac{t-u}{s} \right) dt}{\sqrt{s}} \quad (\text{A})$$

where s is wavelet scale, as above, u is the wavelet translation parameter, and the superscript $*$ represents complex conjugation [18]. The mother wavelet, ψ , represents the kernel, after translation and scaling, against which time-indexed correlations of $f(t)$ are computed to give its wavelet transform. Different choices of ψ provide wavelet transforms with different properties.

Where the Fourier transformation of a CF signal when plotted as a spectrogram yields a peak at CF, a wavelet transformation of the same signal yields a scalogram with a ridge at the cardiac wavelet scale, termed $s_{\heartsuit}$ in this paper (Fig J). In this paper, filtering by $s_{\heartsuit}$ at inverse wavelet transformation is implied, where "filtering" means nullification of all wavelet coefficients other than those for $s_{\heartsuit}$ prior to inverse transformation.

B Library Acceptance Testing

The source code employed here for wavelet vascular pulse wave (PW) cine imaging is written in the Wolfram language and leverages the Mathematica 11 computational library. In this paper, computational experiments with simulated data identify a need for wavelet transforms with a range of frequency and temporal resolution. The Mathematica environment supplies some complex-valued wavelet ψ functions that offer a range of temporal and frequency resolution, including the *GaborWavelet*[] and *PaulWavelet*[] functions. I find that I obtain comparable results with these two ψ families but I employ *GaborWavelet*[] for this analysis because the mathematical structure of the Gabor ψ family explicitly treats the roles of temporal and frequency resolution.

In this paper, transformation by a high temporal resolution wavelet ψ , symbolized by the over hat symbol $\hat{\psi}$, is reified by the library function *GaborWavelet*[1], and transformation by a high frequency resolution wavelet ψ , symbolized by the over tilde symbol $\tilde{\psi}$, is reified with equal performance by the library functions *GaborWavelet*[6] and *GaborWavelet*[12]. The real and imaginary components of *GaborWavelet*[1] and *GaborWavelet*[6] are depicted in Fig K.

The source code for the wavelet functions is not released by the Mathematica library, but computational testing of these functions against test data discloses that *GaborWavelet*[1] behaves as having the values $n = 1$ and $\alpha = 0.21$ and *GaborWavelet*[6] behaves as having the values $n = 6$ and $\alpha = 0.97$ in

$$\psi(t) = \frac{1}{\sqrt[4]{\pi}} e^{\frac{n}{\alpha} i t} e^{-\frac{1}{2}(\frac{t}{\alpha})^2}. \quad (\text{B})$$

C Simulated Angiography Data

A Two-Dimensional Simulation

Motion alias was notable in the human brain surface optical data where the longitudinally orientated vessels have longitudinally oriented bimodality of CF phase. The surface geometry of the human optical angiography facilitated the creation of simulated data. File names of the simulated optical angiograms as supplied as Supporting Information Videos are listed in Table D.

B One-Dimensional Simulation

The performance of high frequency and high temporal resolution wavelet transforms offered by this library can be characterized with simulated one-dimensional data. Two mathematical sinusoids are generated that have frequency, sample rate, and sample length similar to those measure from human optical angiography data, and a frequency one component to simulate bolus passage is added to one of them. Gaussian noise is added to both. The wavelet scalograms show that the bolus passage is over-represented in the high temporal resolution wavelet transform (Fig L.c) since it does not have a ridge at the simulated cardiac frequency.

The respiratory frequency is closer to CF than the bolus passage frequency, so it might be more prone to injecting frequency alias via the high temporal resolution wavelet transforms that are used pixel-wise. Further research with simulated and physiological data might shed light on the extent to which the respiratory component injects frequency alias and to what extent the high frequency resolution wavelet transform that is the cross-correland succeeds in mitigating it. The addition of respiratory frequency phenomena into the simulation is an example of ways to advance the usage of simulated angiography for improving time-frequency resolution strategies.

C Cross-Correlated Wavelets

For two one-dimensional time signals $g(t)$ and $f(t)$, the cross-correlation $h(t)$ is given by

$$h(t) = \int_{-\infty}^{\infty} f(\tau) g(t + \tau) d\tau. \quad (\text{C})$$

If $g(t)$ and $f(t)$ have wavelet transforms $\bar{g}(t)$ and $\bar{f}(t)$, then

$$\bar{h} = \bar{f}^* \bar{g} \quad (\text{D})$$

gives the wavelet transform, $\bar{h}$, of the cross-correlation $h(t)$, where as before the superscript $*$ denotes complex conjugation [19, 20].

Using the notation of this paper (Table C), the proposed reconstruction by wavelet cross-correlation is thus

$$\hat{x} = \hat{c} \tilde{C}^* \quad (\text{E})$$

where $\tilde{C}^*$ is the one-dimensional motion signal wavelet transformed with a wavelet ψ . The cross-correlated result $\hat{x}$ has a hat over symbol $\hat{}$ rather than the unspecified wavelet resolution as $\bar{x}$ to specify that at inverse wavelet transformation a high temporal resolution wavelet ψ will be employed (with filtering for $s_{\heartsuit}$).

Optionally, $\tilde{C}$ may be replaced by the normalized

$$\frac{\tilde{C}}{|\tilde{C}|} \quad (\text{F})$$

to enhance comparability between subjects and species.

The wavelet cross-correlation of Equation E may be simulated as in the method of Fig L with a further cross-correlation step between the high frequency and high temporal resolution wavelet transforms (Fig M). To evaluate phase resolution, the test signals are 1/2 cycle apart.

The simulation indicates that some loss of frequency resolution when employing a high temporal resolution wavelet ψ can be mitigated by cross correlation with the transform with a high frequency resolution wavelet ψ (Fig Mc). There remains a role for second order phase correction after cross-correlation, as shown by the drift of phase below 0. in Fig Mh. With both the human and piglet data below, a second order phase correction is applied to bring the mean phase of the high resolution data in line with that of the high frequency resolution data.

The application of Equation E to pixel-wise time signals, filtering for $s_{\heartsuit}$ and pixel-wise inverse wavelet transformation gives the complex-valued vascular PW cine imaging proposed in this paper. It may be rendered with magnitude as brightness and phase as hue per the color model described in Fig H to give cine CF images, with example snapshots shown in Fig 5 of the main document. The video files of the simulated optical angiograms and some test reconstructions are supplied as S40Video through S52Video.

Other signals, intracranial or extracranial, could be selected to serve as the metronomic high frequency resolution signal for cross-correlation in Equation E. The choice of $\tilde{C}$ and other aspects of the CF angiographic phenomena reconstruction method presented here of course may be revised and improved based on further research. In addition, the use of higher acquisition rate angiographic methods than those employed here should permit the shift of both the high temporal resolution and high frequency resolution wavelet ψ s in the direction of greater frequency resolution, potentially mitigating the frequency alias associated with the use of high temporal resolution wavelet ψ s.

D Time Signal Norming Of Wavelet Signal

The wavelet transforms may introduce other artifacts. At the ends of each time signal a wavelet transform may introduce a signal wrap artifact if the last signal intensity value does not match the first. In addition, the use of a high frequency resolution wavelet ψ may cause an otherwise sharp time signal to be spread locally. These artifacts introduced by the wavelet transforms may be mitigated by point-wise multiplication ("norming") of the wavelet filtered signal by the original, averaged time signal (Fig N).

F Wavelet Angiography Results

Snapshot vascular PW images for subjects h2-4 and p2-3 are presented in Fig O.

Even though the PW images reconstructed from piglet ultrasound have a more grainy appearance than those from human optical angiography, there is greater cardiac frequency signal in the piglet ultrasound images. This may be seen by comparing the cardiac frequency SNRs in the piglet ultrasound versus human optical angiographic data (compare SNRs in Tables A and B). The bases of these differences remain unknown.

G Raw Data And Wavelet Angiography Video Files

All raw data and computationally reconstructed video data are supplied as Supporting Information. The video file names for the raw data and for the key vascular PW cine images are presented in Table E.

H Angiographic Time Of Flight And Arteriovenous Pixel Classification

With the optical technique, the infrared filter provides a black background prior to the arrival of ICG whereas with the ultrasound technique the background consists of a non-blank background (an anatomic image) prior to the perflutren arrival and thus requires a background filter. Selecting the phase of the Fourier frequency one component removes the background, which is contained in the frequency zero component, providing a single method for estimating angiographic time of flight (ATOF) that works equivalently for both the human and piglet data. For the human data, where the background is black, ATOF may be estimated by other methods such as mean arrival time

$$\frac{\sum_t t c_{i,j,t}}{\sum_t c_{i,j,t}}. \quad (G)$$

Scattergrams of the phase of the frequency one component versus mean arrival time show a linear relationship for all human subjects. The variability in signal between pixels for the ultrasound data before perflutren arrival does not permit such a comparison.

The ATOF histograms and arteriovenous classified time intensity curves for subjects h2-4 and p2-3 are presented in Fig P.

In using a simple cutoff in the ATOF histogram to separate arterial from venous pixels must produce significant group overlap with pixels classified as venous having an arterial component and vice versa. However, the time intensity curves after the arterial versus venous classification have shapes broadly consistent with each being respectively a primarily arterial and primarily venous pixel population (second column of Figure P of this document and Figure 7 of the main document). The time intensity curves with the expected arterial and venous temporal profile suggests that the arterial versus venous classification performed here is sufficient to examine trends in how the two groups differ in PW phase distribution.

I Time-Indexed Phase Histogram

A Pixels With Lower Signal Are Excluded From The Phase Histograms

In the spatiotemporal grid of a reconstruction of x , a cumulative distribution function is constructed of the magnitude of the complex-valued grid elements. The shape of the curves shows that about 20% of the grid elements have significant CF signal for both the human and piglet subjects. For constructing the phase histograms the phases are drawn from the grid elements in the upper 20% of CF magnitude (Fig Q). Separate phase histograms are made of the arterial and venous classified pixels, then the two are merged into one (Fig R).

B Composite Arteriovenous Time-Indexed Phase Histogram

Wavelet cine angiograms are analyzed for the distribution of phase to produce the time-indexed phase histograms. The classification of pixels by ATOF is used to produce a composite arteriovenous time-indexed phase histogram. Such a histogram has two component time-indexed-phase histograms, an arterial one depicted in red and a venous one depicted in blue. The construction method is depicted in Fig R.

The time-indexed phase histograms with selected line scans for subjects h2-4 and p2-3 are presented in Fig S. These data suggest the presence of a consistent arterial versus venous phase difference.

The component arterial and venous phase histograms depend on the binary versus venous classification of pixels based on ATOF. The arterial and venous phase histograms are influenced by the statistical properties of the arterial versus venous classification. Without further research, no conclusions should be drawn from the comparative shapes of adjacent arterial and venous phase histogram data.

J Motion-Referenced Time-Indexed Phase Histogram

The motion-referenced wavelet cine angiograms are analyzed for the distribution of motion-referenced phase to produce these. The motion-referenced time-indexed phase histograms with collapsed circular phase histograms for subjects h2-4 and p2-3 are presented in Fig T. The difference between arterial and venous phase appears more fixed than the difference between either and pulse motion phase. The summary circular phase histograms all show a difference between arterial and venous phase but the phase differences and histogram shapes are different between subjects. The basis of this merits further research.

Human Subject ID	h1	h2	h3	h4
Aneurysm Location	MCA	MCA	AComm	MCA
Heart Rate (Hz)	.85	.98	.87	1.19
Sample Rate (Hz)	30.	30.	30.	30.
Sampling Period (sec)	55	29.3	51.6	38.6
Motion SNR	30.8	6.5	4.5	8.4
Angiographic SNR	5.4	3.9	2.3	1.6

Table A. Human Optical Angiography Summary. Sample Rate and Heart Rate are given in Hertz. MCA=middle cerebral artery, AComm=anterior communicating cerebral artery.

Piglet Subject ID	p1	p2	p3
Image Dimensions	360×342	393×420	418×418
Intracranial Pixels	66%	87%	72%
Heart Rate (Hz)	1.5	2.6	2.8
Sample Rate (Hz)	30.	30.	10.
Sampling Period (sec)	21.7	11.3	14.0
Motion SNR	4.5	14.5	9.9
Angiographic SNR	19.6	13.5	6.

Table B. Piglet Cranial Window Ultrasound Summary. Image Dimensions are measured in pixels, at 340 pixels/4 cm 84 pixels/cm. The intracranial pixels are defined by the respective intracranial polygon.

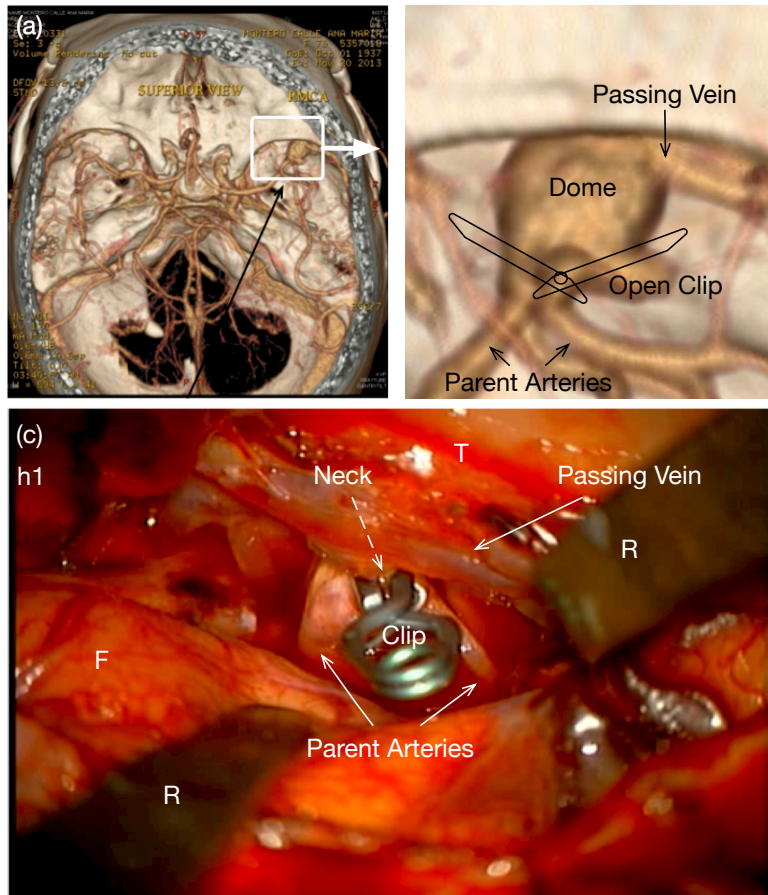

Fig A. Right Middle Cerebral Artery (MCA) Aneurysm (Subject h1). (a) 3D rendering from CTA of R MCA aneurysm with (b) magnified view showing the parent arteries, the aneurysm dome, and the proposed aneurysm clip position. (c) Frozen frame from the visible video track obtained after aneurysm clipping shows the aneurysm neck between the clip blades and apparent sparing of the parent vessels, circumstances to be verified with intraoperative angiography.

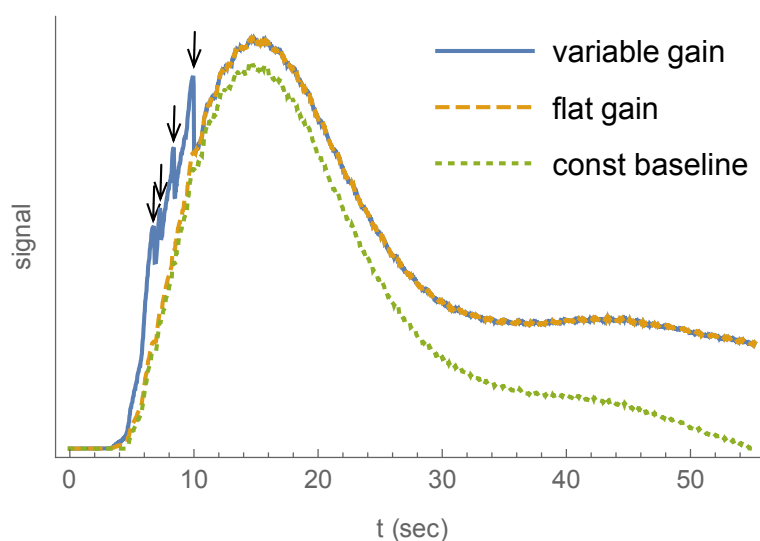

Fig B. Removal Of Signal Gain Adjustment. The Pentero Micro-scope ICG hardware automatically reduces the gain to prevent saturation of the image when the total signal intensity increases sharply (gain reductions shown by arrows in this example). At analysis, the gain reductions are semi-automatically identified, measured and reversed to yield flat gain for further analysis.

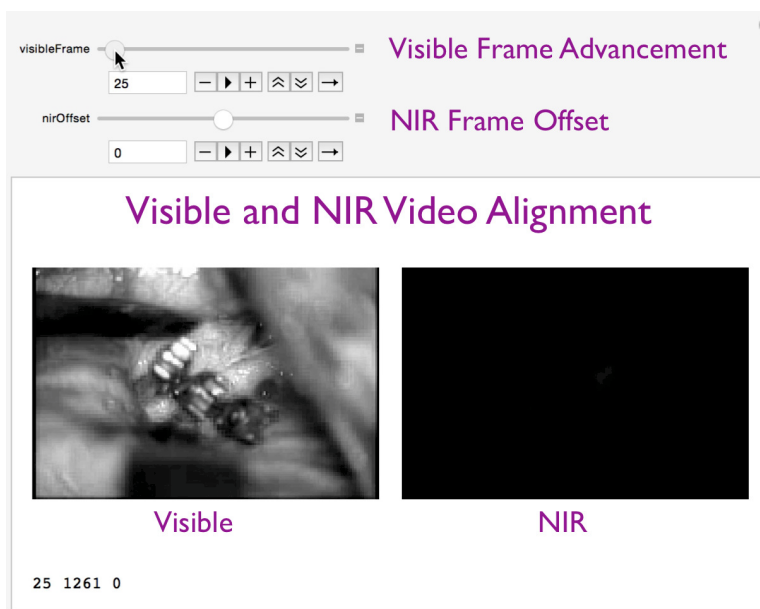

Fig C. Visible And NIR Video Synchronization. An interactive manipulation program is used to find the relative video frame offset needed to synchronize the visible and NIR videos. Annotations are in purple. An edited video demo is supplied as Supporting Information Video S52.

Human Optical Brain Surface Angiography

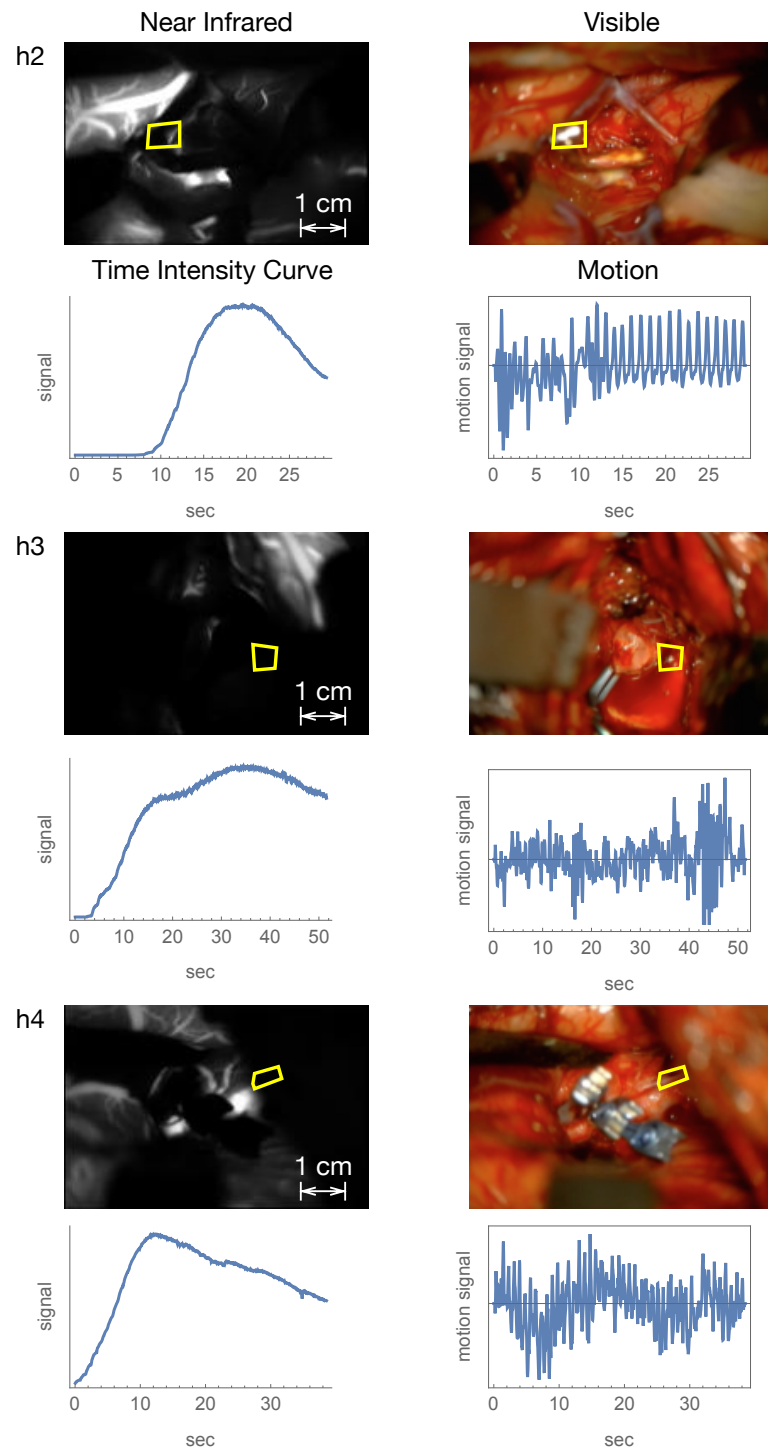

Fig D. Human Brain Surface Optical Angiography. Representative snapshot images and temporal data summaries for human subjects h2-4. The yellow polygon in the images gives the region for motion tracking.

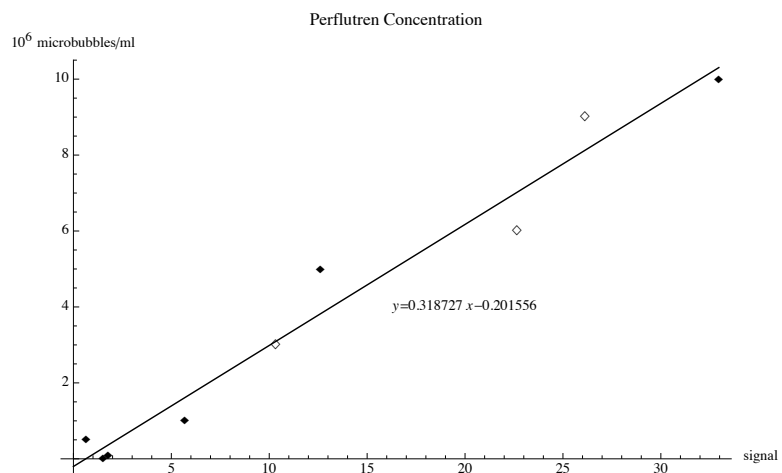

Fig E. Ultrasound Signal Intensity Versus Perflutren Concentration. Data are combined from the open-loop (◇) and closed-loop (◆) phantom measurements. In a flowing phantom, perflutren has an approximately linear signal to concentration relation. For the linear regression $r = 0.98$, $p < 0.0001$.

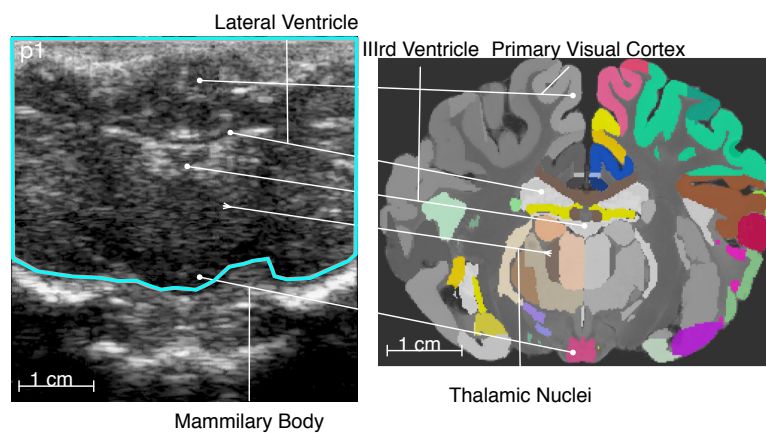

Fig F. Piglet Cranial Ultrasound Image With Matching Piglet Atlas Slice. The cyan polygon defines the intracranial region. A matched coronal-plane image from a pig brain anatomical atlas as rendered by 3D Slicer version 4.3.0.

Piglet Cranial Window Ultrasound Angiography

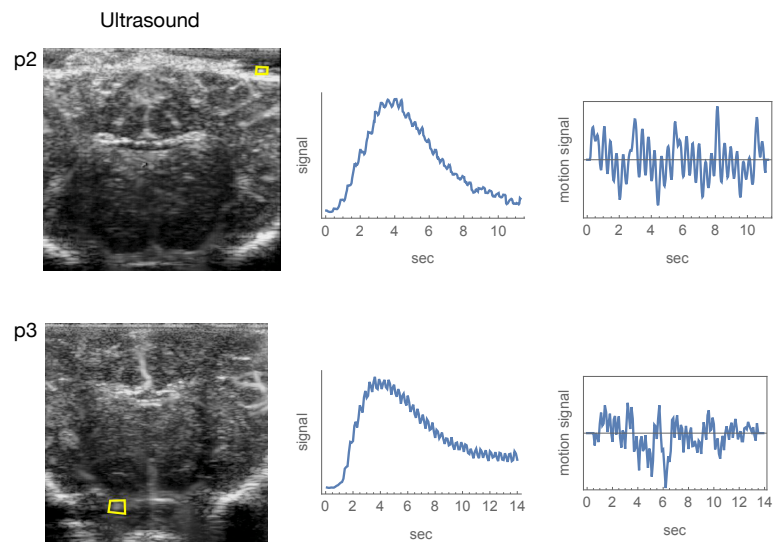

Fig G. Piglet Ultrasound Angiography. Representative snapshot images and temporal data summaries for piglet subjects p2-p3. The yellow polygon in the images gives the region for motion tracking.

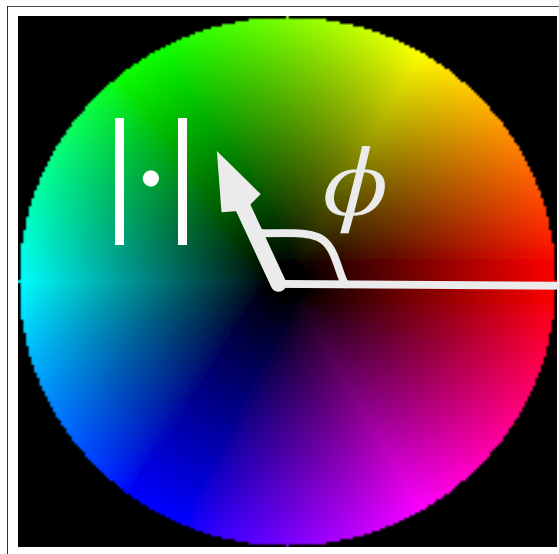

Fig H. Hue-Brightness Model For Rendering A Pixel From Complex Datum. To represent a complex-valued datum as a pixel in an image, distance from the origin in the complex plane ($| \cdot |$) is rendered as brightness and phase (ϕ) as hue. In this paper, the rendering model may be represented mnemonically in figures by the icon on the right.

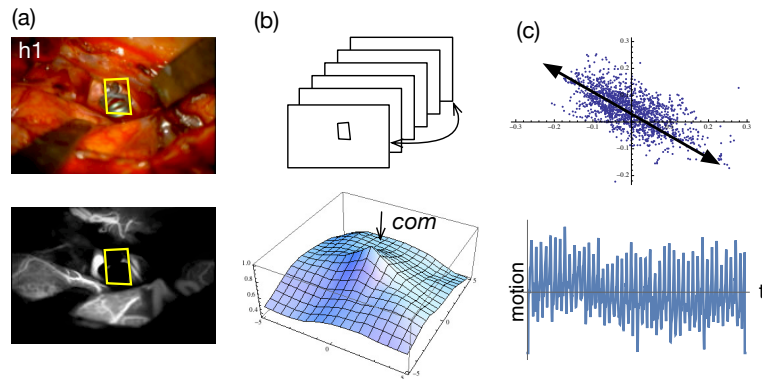

Fig I. Brain Motion Signal From Human Optical Data. (a) Brain pulse motion is measure in a region of interest (yellow polygon). (b) The correlation matrix for an array of displacements is computed for each frame pair 5 frames apart. The center of mass (com) of the matrix is taken to estimate the 2-dimensional displacement between the frame pair. (c) A scattergram of the displacements with major axis (arrow). Time-Indexed projections of the 2-D displacements along the major axis give the motion signal.

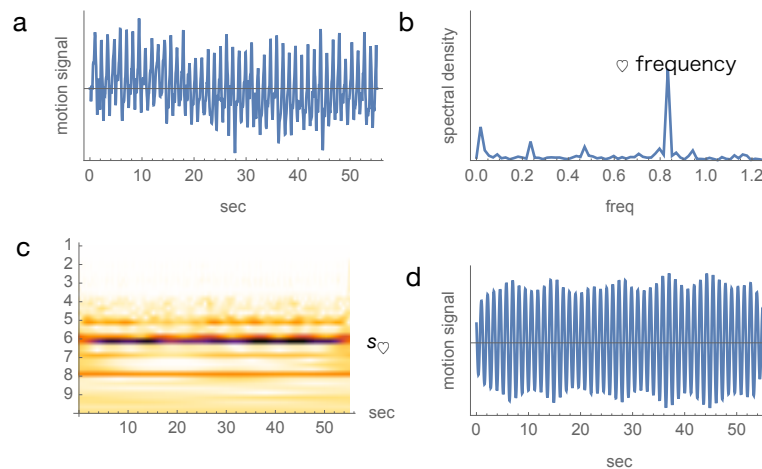

Fig J. Cardiac Frequency And Wavelet Scale. A wavelet transform may offer a time-resolved way of analyzing CF phenomena. Fourier analysis of the (a) motion signal for subject h1 results in (b) a spectral density peak at CF. This is not time-resolved. (c) Wavelet transformation results in a time-indexed ridge at cardiac wavelet scale, $s_{♡}$, which approximately corresponds to cardiac period. (d) Inverse wavelet transformation of (c) with filtering by $s_{♡}$ yields a smoothed, time-resolved pulse motion signal.

Symbol	Definition	Example/Comment
$ $	magnitude of complex number	$ x $
ϕ	phase of complex number	ϕ_x
subscript i,j	i,j^{th} pixel	commonly implied
lower case	pixel-wise	$c_{i,j}$ = time intensity curve
upper case	frame-wise	M = motion signal
ψ	wavelet mother function	choose complex-valued
$s_{\heartsuit}$	wavelet scale for CF	filter for inverse wavelet transform
c	time intensity curve indexed for pixel	$c_{i,j}$
C	aggregate time intensity curve	$\tilde{C}$
M	motion signal	$\tilde{M}$
over bar $\bar{}$	wavelet transform	$\bar{c}_{i,j}$
over hat $\hat{}$	high temporal resolution wavelet transform	$\hat{c}_{i,j}$
over tilde $\tilde{}$	high frequency resolution wavelet transform	$\tilde{M}$
superscript $*$	complex conjugate	$\tilde{M}^*$
$\hat{x}$	vascular PW cine imaging by cross-correlation of $\hat{c}$ with M	$\hat{x} = \hat{c} \tilde{M}^*$
x	inverse HTR wavelet transform of $\hat{x}$	$s_{\heartsuit}$ filtered, complex-valued
$\hat{y}$	motion-referenced vascular PW cine imaging	$\hat{y} = \hat{c} \tilde{C}^* \tilde{M}$

Table C. Notation. Sampling by uniformly spaced time is implied.

Simulation	File	$\hat{c}$	$\tilde{c}$	x
5*Motion	S40 Video			
Motion	S41 Video	S42 Video	S43 Video	S44 Video
Circulation	S45 Video	S46 Video	S47 Video	S48 Video
Circulation & Motion	S49 Video	S50 Video	S51 Video	S52 Video

Table D. Simulations. Based on human subject h1. The video files are supplied as Support Information. The right 3 column headings give the wavelet reconstruction method.

Subject	Visible	Angiogram	$\hat{c}$	$\tilde{c}$	x	y
h1	S1 Video	S2 Video	S3 Video	S4 Video	S5 Video	S6 Video
h2	S7 Video	S8 Video	S9 Video	S10 Video	S11 Video	S12 Video
h3	S13 Video	S14 Video	S15 Video	S16 Video	S17 Video	S18 Video
h4	S19 Video	S20 Video	S21 Video	S22 Video	S23 Video	S24 Video
p1	S25 Video	NA	S26 Video	S27 Video	S28 Video	S29 Video
p2	S30 Video	NA	S31 Video	S32 Video	S33 Video	S34 Video
p3	S35 Video	NA	S36 Video	S37 Video	S38 Video	S39 Video

Table E. Video Data. The right 4 column headings allude to the wavelet reconstruction method. The column header $\hat{c}$ indicates high temporal resolution wavelet transform, $\tilde{c}$ indicates high frequency resolution wavelet transform, x indicates cross-correlated wavelet transform, and y indicates motion-referenced cross-correlated wavelet transform,.

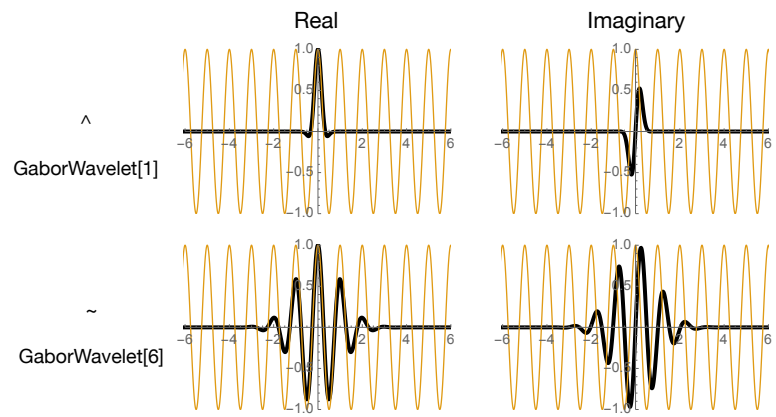

Fig K. Temporal And Frequency Resolution Of A Wavelet ψ Function.

Vertical axis is magnitude and horizontal axis is time. The narrow temporal span of the real and imaginary components of the upper row corresponding to the function call in this wavelet computational library of *GaborWavelet[1]* reflects a high temporal but low frequency resolution of that wavelet ψ . The broad temporal span corresponding to the function call *GaborWavelet[6]* reflects a low temporal but high frequency resolution.

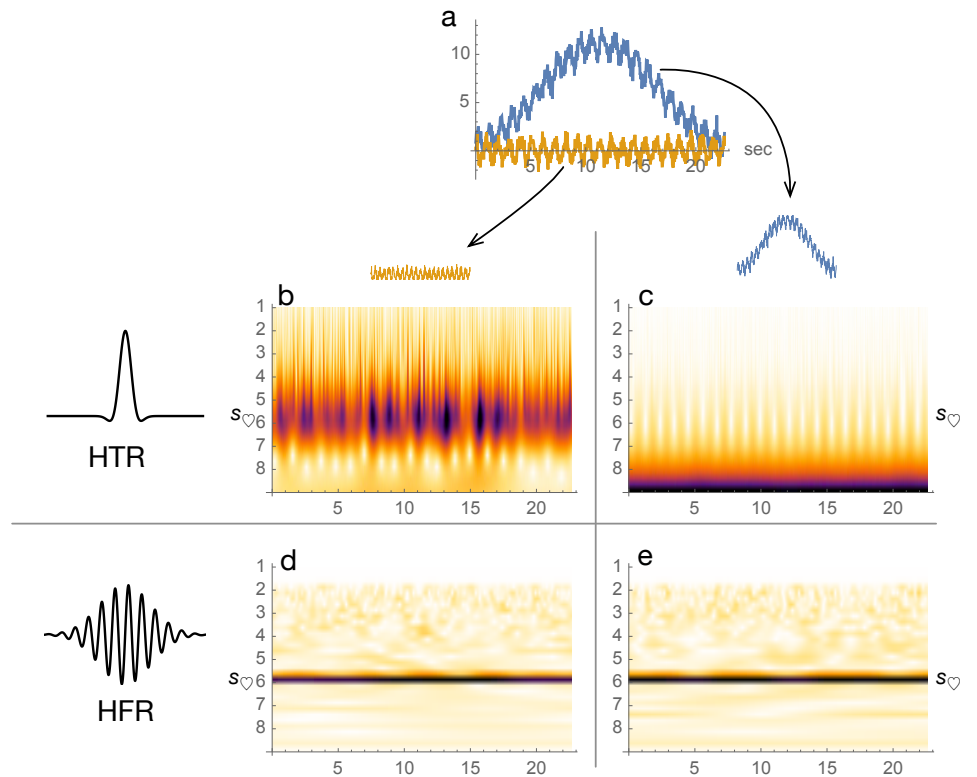

Fig L. Simulated One Dimensional Data. The vertical axes of the scalograms are wavelet scale and the horizontal axes are time in seconds. (a) Simulated angiographic intensity curves with CF variation (orange) and bolus passage (blue). (b,c) Scalograms from high temporal resolution (HTR as computed by *GaborWavelet*[1]) ψ show blunting or loss of ridge at s_0 . (d,e) The ridge at s_0 is preserved in the high frequency resolution (HFR as computed by *GaborWavelet*[12]) ψ scalograms, but not in the high temporal resolution ψ scalograms, which indicates the presence of frequency aliasing. This simulation shows that use of a pure HTR wavelet ψ may lead to excessive frequency alias in reconstructed images.

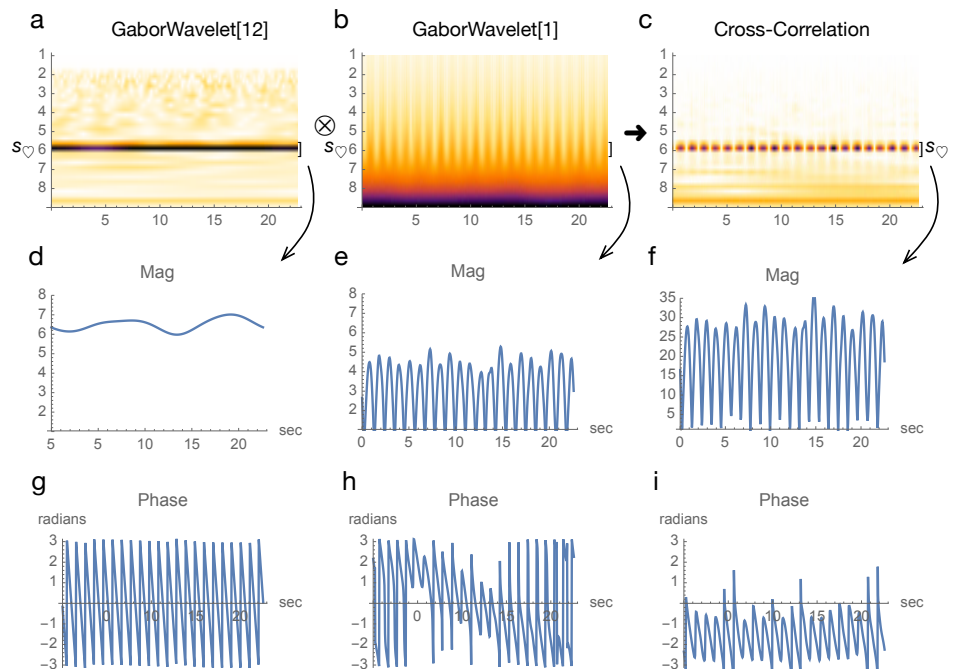

Fig M. Cross-Correlated Wavelet Simulation. The vertical axes are wavelet scale and the horizontal axes are time in seconds. In the employed wavelet computational library, the function *GaborWavelet*[1] is a high temporal resolution wavelet transform and *GaborWavelet*[12] a high frequency resolution transform. Graph (c) is the cross-correlation of (a) and (b). The magnitude and phase filtered by cardiac wavelet scale are in the second and third rows. This one-dimensional simulation suggests that cross-correlation of a high temporal resolution wavelet transform by a high frequency resolution wavelet transform can attenuate some of the frequency alias that accompanies a high temporal resolution wavelet transform alone.

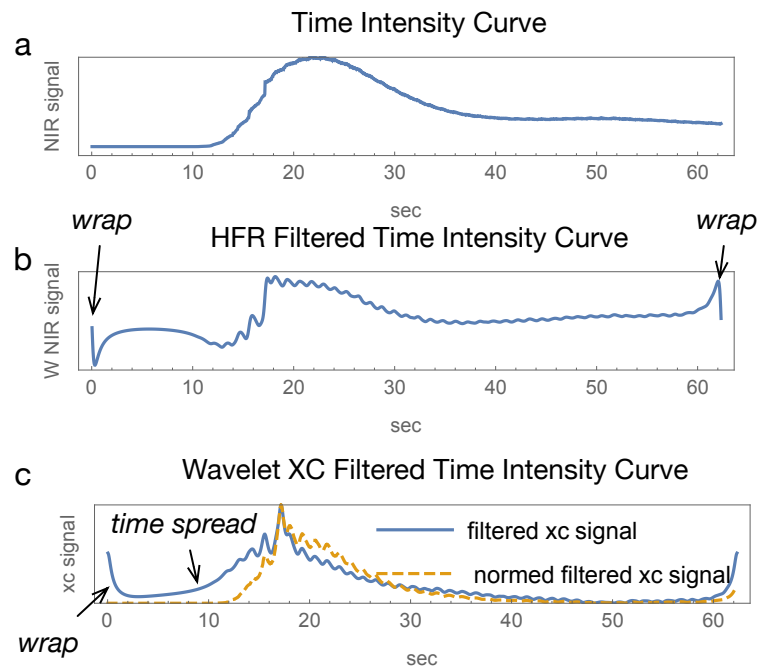

Fig N. Signal Norming To Mitigate Time Spread And Phase Wrap Artifacts. Filtering of (a) the NIR time intensity curve $C(t)$ for $s_\heartsuit$ by a high frequency resolution wavelet ψ produces (b) wrap artifact. (c) The inverse wavelet transform of the cross-correlated (XC) signal has wrap and time spread artifacts, which are mitigated by signal norming by (a).

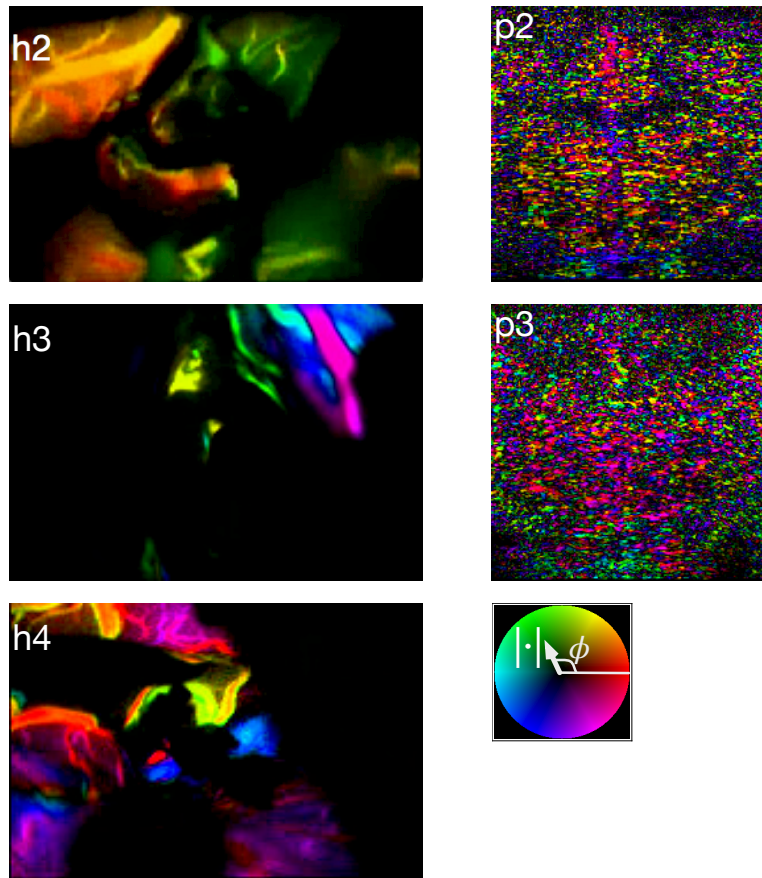

Fig O. Wavelet Angiography Snapshots. Images are selected from the vicinity of the peak of the angiographic time intensity curve.

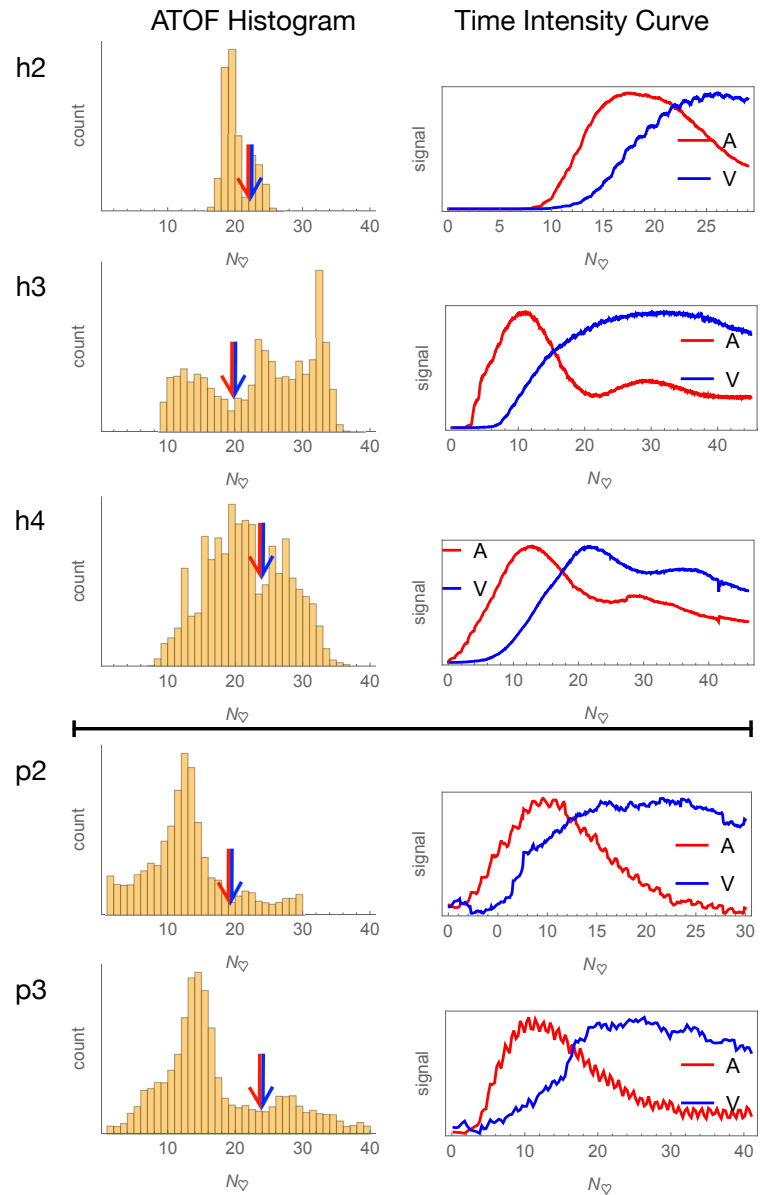

Fig P. Arteriovenous Classification By ATOF Histogram Analysis. ATOF is measured in heartbeats. The derived time intensity curves are consistent with predominantly arterial and venous temporal behavior.

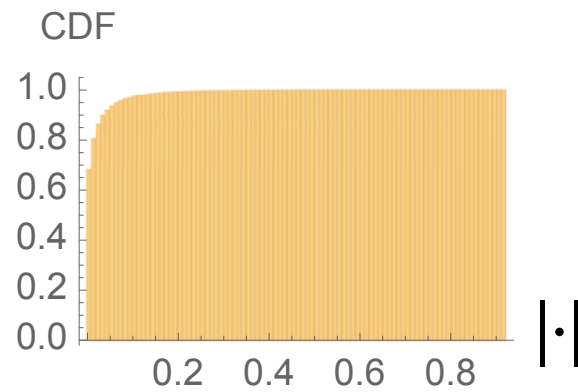

Fig Q. Empiric Cumulative Distribution Function Of CF Magnitude. Each complex-valued datum is expressed in polar coordinates as magnitude $|\bullet|$ and phase ϕ . A CDF is made of the magnitudes and only those ϕ values with $|\bullet|$ greater than the 80th percentile are included in the phase histograms. This reduces the contribution by phase values in low signal pixels. This figure is for h1. Those for the h2-4 and p1-3 are similar in shape.

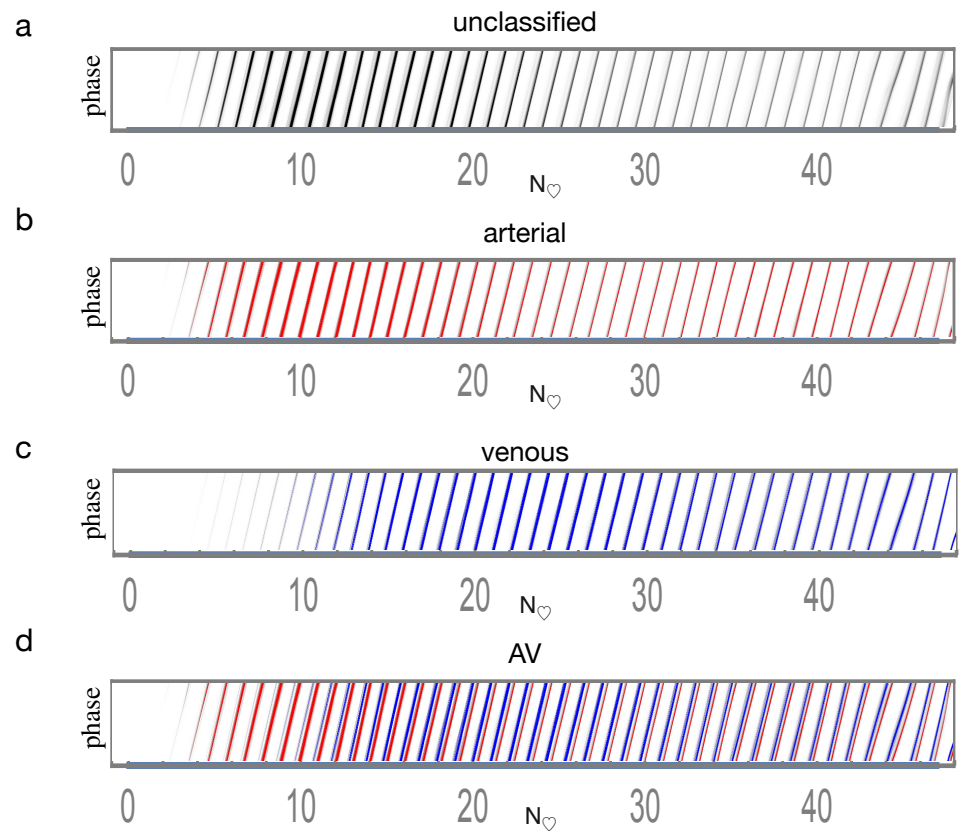

Fig R. Construction Of A Time-Indexed Phase Histogram. An illustrative (a) overall time-indexed phase histogram is made before arterial versus venous binary classification. The pixels are classified as arterial versus venous by ATOF and separate (b) and (c) time-indexed phase histograms are made of each. The arterial and venous time-indexed histograms are (d) superimposed to compare the arterial and venous phase patterns.

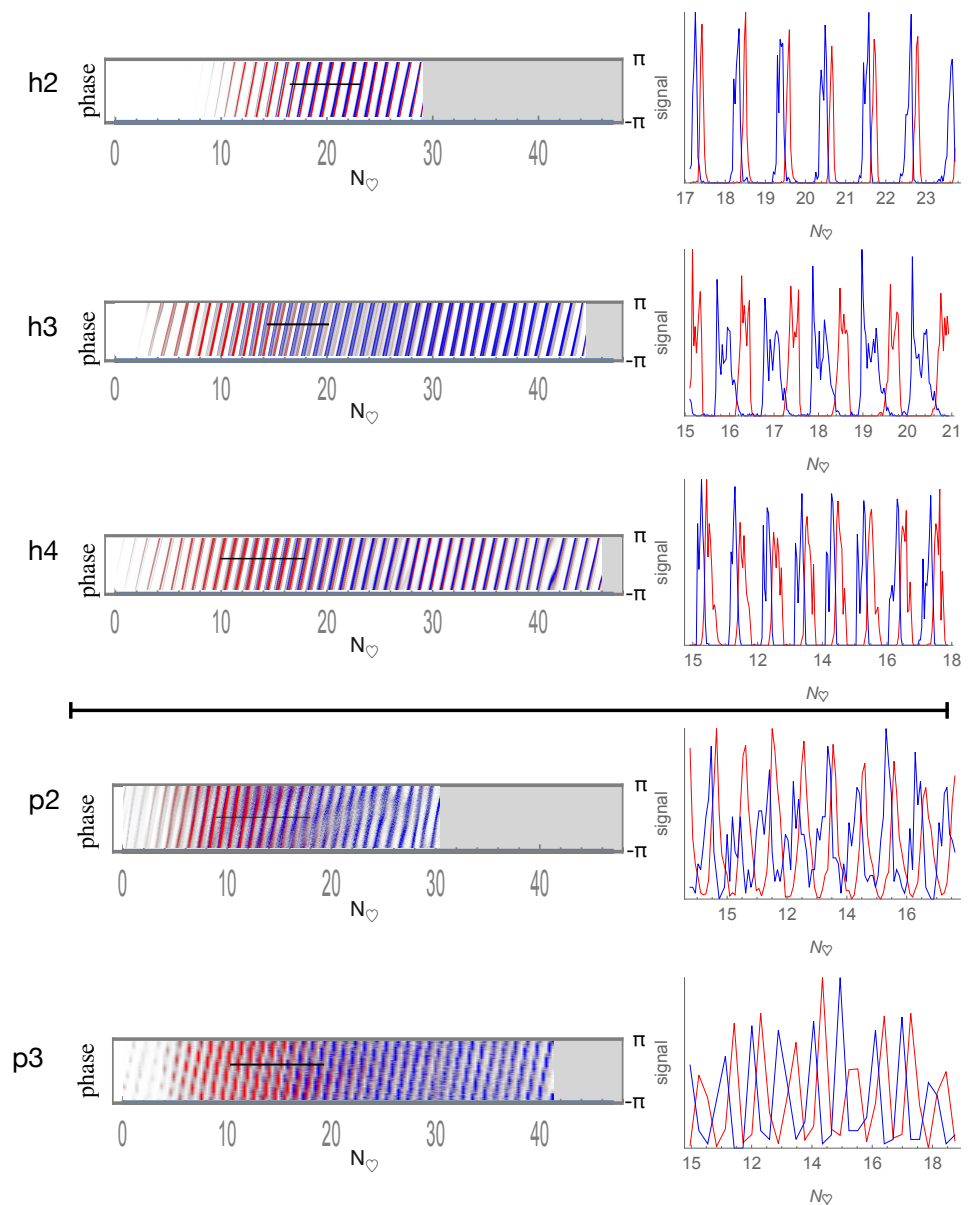

Fig S. Time-Indexed Phase Histograms With Line Scans. Pixels classified by ATOF as arterial are in red and those as venous in blue. Relative histogram count is represented as brightness. The horizontal axis is time is depicted in heartbeats. The vertical axis is phase ranging as $[-\pi, \pi]$. In all subjects there is a consistent arteriovenous phase difference across the bolus passage.

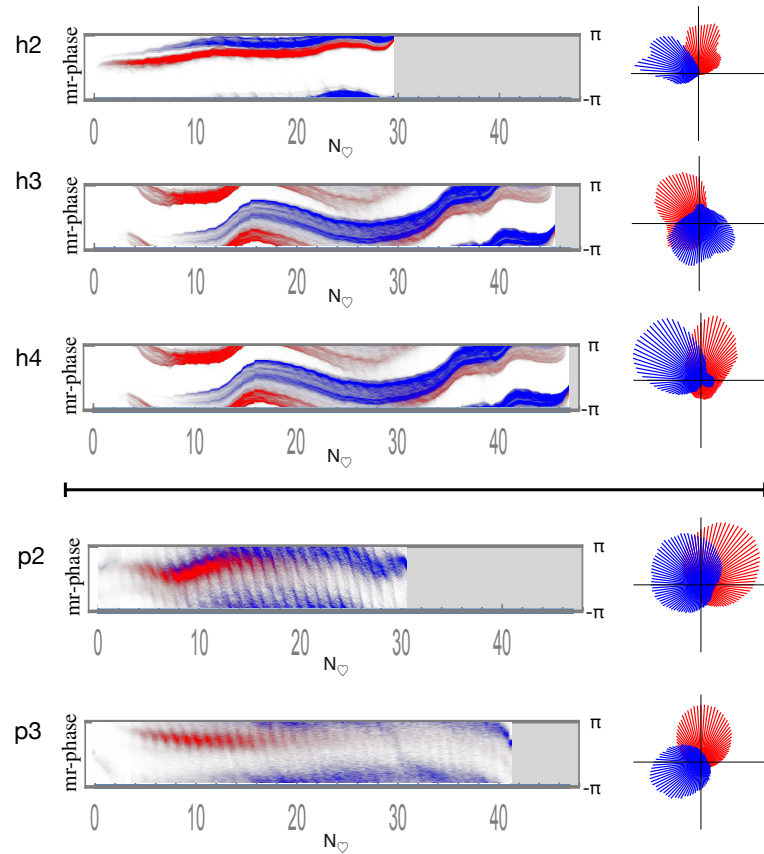

Fig T. Motion-Referenced Time-Indexed Phase Histograms With Aggregate Circular Phase Histograms. Pixels classified by ATOF as arterial are in red and those as venous in blue. Relative histogram count is represented as brightness. The horizontal axis is time is depicted in heartbeats to facilitate human-piglet comparison. The vertical axis is phase ranging as $[-\pi, \pi]$. Arterial versus venous phase appears approximately fixed in difference across the entire bolus passage. The circular phase histograms on the right are calculated by summing the time-indexed phase histograms on the left across time.

References

1. Lawton MT. Seven Aneurysms: Tenets and Techniques for Clipping. New York: Thieme; 2011.
2. Organization IS. Multimedia Formats. *http://standards.iso.org/ittf/PubliclyAvailableStandards/c051533_ISO_IEC_4496-12_2008.zip*; 2008.
3. Meairs S, Alonso A. Ultrasound, microbubbles and the blood-brain barrier. *Progress in Biophysics and Molecular Biology*. 2007;93(1-3):354–362.
4. Platts DG, Fraser JF. Contrast echocardiography in critical care: echoes of the future? A review of the role of microsphere contrast echocardiography. *Critical Care and Resuscitation*. 2011;13(1):44–55.
5. Ekroll IK, Swillens A, Segers P, Dahl T, Torp H, Lovstakken L. Simultaneous quantification of flow and tissue velocities based on multi-angle plane wave imaging. *IEEE Transactions on Ultrasonics, Ferroelectrics, and Frequency Control*. 2013;60(4):727–738.
6. Gauthier M, Tabarout F, Leguerney I, Polrot M, Pitre S, Peronneau P, et al. Assessment of quantitative perfusion parameters by dynamic contrast-enhanced sonography using a deconvolution method: an in vitro and in vivo study. *Journal of Ultrasound in Medicine*. 2012;31(4):595–608.
7. Gauthier TP, Averkiou MA, Leen ELS. Perfusion quantification using dynamic contrast-enhanced ultrasound: the impact of dynamic range and gain on time-intensity curves. *Ultrasonics*. 2011;51(1):102–106.
8. Kogan P, Johnson KA, Feingold S, Garrett N, Guracar I, Arendshorst WJ, et al. Validation of dynamic contrast-enhanced ultrasound in rodent kidneys as an absolute quantitative method for measuring blood perfusion. *Ultrasound in Medicine & Biology*. 2011;37(6):900–908.
9. Strouthos C, Lampaskis M, Sboros V, McNeilly A, Averkiou M. Indicator dilution models for the quantification of microvascular blood flow with bolus administration of ultrasound contrast agents. *IEEE Transactions on Ultrasonics, Ferroelectrics, and Frequency Control*. 2010;57(6):1296–1310.
10. Thijssen JM, de Korte CL. Modeling ultrasound contrast measurement of blood flow and perfusion in biological tissue. *Ultrasound Med Biol*. 2005;31(2):279–285.
11. van Wijk MC, Klaessens JHGM, Hopman JCW, Liem KD, Thijssen JM. Assessment of local changes of cerebral perfusion and blood concentration by ultrasound harmonic B-mode contrast measurement in piglet. *Ultrasound in Medicine & Biology*. 2003;29(9):1253–1260.
12. Nillesen MM, Lopata RGP, de Boode WP, Gerrits IH, Huisman HJ, Thijssen JM, et al. In vivo validation of cardiac output assessment in non-standard 3D echocardiographic images. *Phys Med Biol*. 2009;54(7):1951–1962.
13. Zhao X, Zhong H, Wan M, Shen L. Ultrasound Contrast Imaging Based on a Novel Algorithm Combined Pulse Inversion with Wavelet Transform. *Ultrasound in Medicine & Biology*. 2011;37(8):1292–1305.

-
14. Provost J, Papadacci C, Arango JE, Imbault M, Fink M, Gennisson JL, et al. 3D ultrafast ultrasound imaging in vivo. *Physics in Medicine and Biology*. 2014;59(19):L1.
  15. Gauthier TP, Chebil M, Peronneau P, Lassau N. In vitro evaluation of the impact of ultrasound scanner settings and contrast bolus volume on time-intensity curves. *Ultrasonics*. 2012;52(1):12–19.
  16. Lassen NA, Perl W. *Tracer Kinetic Methods in Medical Physiology*. Raven, New York; 1979.
  17. Smith BC. In: *Fundamentals of Fourier Transform Infrared Spectroscopy*, Second Edition. CRC Press; 2011. p. 14. Available from: https://books.google.com/books?id=LR9HkK2cP_0C.
  18. Daubechies I. *Ten Lectures on Wavelets*. Society for Industrial and Applied Mathematics; 1992.
  19. Klimenko S, Mitselmakher G, Sazonov A. A cross-correlation technique in wavelet domain for detection of stochastic gravitational waves. *General Relativity and Quantum Cosmology*. 2002;.
  20. Serroukh A. Wavelet coefficients cross-correlation analysis of time series. *Electronic Journal of Applied Statistical Analysis*. 2012;5(2):289–296.
